# Lactoferrin-cyanidin-3-glucoside nanoparticles alleviate inflammation and oxidative stress via *Sesn2/Nrf2* activation in mastitis

**DOI:** 10.1016/j.mtbio.2025.102491

**Published:** 2025-10-31

**Authors:** Fructueux Modeste Amona, Yipeng Pang, Xiaohan Chen, Zilu Liu, Chenyang Su, Jiachen Yang, Kejun Liu, Qiyu Wu, Bingbing Liu, Xi Chen, Chunlei Zhang

**Affiliations:** Institute of Cellular and Molecular Biology, School of Life Science, Jiangsu Normal University, Xuzhou, 221116, Jiangsu, China

**Keywords:** Lactoferrin-cyanidin-3-glucoside nanoparticles, Inflammation, Oxidative stress, *Sesn2/Nrf2* signaling, Mastitis

## Abstract

Mastitis, driven by oxidative-inflammatory responses to *S. aureus*, lacks efficient therapies. Recently, protein-anthocyanin encapsulation has shown significant advances in biomedicine, but it faces structural stability/suboptimal efficiency issues, as well as an underexplored ROS-responsive targeting pathway. To address these issues, we engineered lactoferrin-encapsulated cyanidin-3-glucoside nanoparticles (LF-C3GNPs) using electrohydrodynamic nanotechnology for the management of oxidative-inflammatory disorders in mastitis. The NPs exhibited ROS-responsive C3G release, high colloidal stability, and H_2_O_2_-scavenging activity. In LTA- and H_2_O_2_-stimulated HC11 mammary epithelial cells, LF-C3GNPs attenuated inflammation and oxidative damage by activating *Sesn2/Nrf2* signaling, which enhanced antioxidant genes. LF-C3GNPs outperformed dexamethasone (DEX) in *S. aureus*-induced murine mastitis by reducing bacterial load and neutrophil infiltration, decreasing pro-inflammatory cytokines, and restoring redox balance. LF-C3GNPs showed excellent biosafety and hemocompatibility *in vivo*. LF-C3GNPs represent a potent nanomedicine for mastitis via *Sesn2/Nrf2* activation, with clinical translational promise. This study revealed significant anti-inflammatory and antioxidative effects of ROS-scavenging natural product self-assembled nanomedicine, offering a model for eco-friendly, efficient nano-antioxidant development to treat oxidative-inflammatory disorders related to diseases such as mastitis.

## Highlights

• ROS-responsive LF-C3GNPs fabricated by electrohydrodynamic nanoencapsulation show high stability.
• ROS-triggered release enables targeted antioxidant delivery and scavenges H₂O₂ effectively.
• LF-C3GNPs activate Sesn2/Nrf2 and inhibit STAT3/2 to restore redox balance in mastitis.
• Superior to dexamethasone in reducing bacterial load, cytokines, and neutrophil infiltration.
• Excellent colloidal stability, hemocompatibility, and organ safety support clinical translation.

## Graphical abstract

**Figure:**
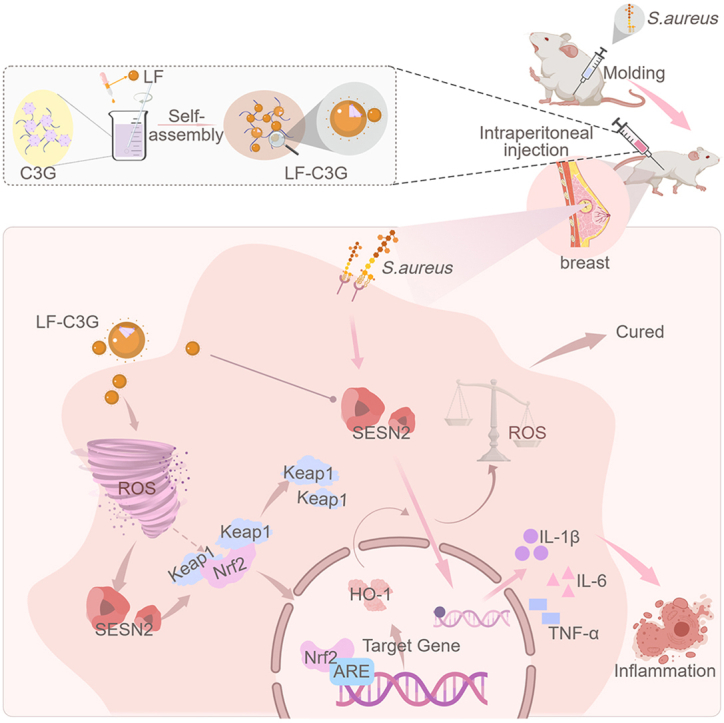

## 1 Introduction

Inflammation is a natural protective response that eliminates harmful stimuli, such as pathogens, physical injuries, or toxins, and restores homeostasis via repair mechanisms [1,2]. However, unresolved inflammation can lead to chronic inflammatory disorders [3,4], including mastitis—a localized inflammatory condition commonly triggered by bacterial infections in mammary tissues during lactation in women and animals [5]. *S. aureus*-induced mastitis, prevalent among postpartum humans and animals, frequently advances to pus-forming stages that compromise the structural stability of the mammary gland. Delayed or inadequate treatment results in systemic infections, increases breast-related disorders, and causes substantial health risks for lactating women and animals [5,6]. Furthermore, *S. aureus* is resistant to numerous conventional antibiotics, and even antibiotic-sensitive strains can still result in severe infections that induce oxidative-inflammatory conditions, critical interconnected mechanisms in the development of mastitis [5]. Notably, oxidative stress is a major factor in mastitis development, caused by an imbalance between mammary antioxidant defenses and excessive reactive oxygen species (ROS) production owing to heightened metabolic activity [7], which can reduce lactation by 3–20 % in women and trigger the inflammatory process. Thus, tailoring phytochemicals to restore redox balance is a promising therapeutic strategy for managing inflammatory diseases such as mastitis.

Anthocyanins, unique natural polyphenolic phytochemicals and abundant water-soluble pigments in fruits and vegetables, have gained substantial interest due to their diverse health benefits [8,9]. Notably, Cyanidin-3-O-glucoside (C3G), a major anthocyanin, is widely researched in biomedicine and food science due to its potent antioxidant, anti-inflammatory, and antimicrobial properties [10,11]. Unlike curcumin and epigallocatechin gallate (EGCG) [12,13], C3G offers unique advantages, including widespread availability, synergistic modulation of redox pathways, and excellent safety properties even at high doses. Its metabolic conversion by the gut microbiota into bioactive phenolic derivatives, such as protocatechuic acid (PCA), phloroglucinaldehyde (PGA), vanillic acid (VA), and ferulic acid (FA), enhances systemic antioxidant and anti-inflammatory effects, making it ideal for mastitis [14]. However, C3G's potential applications are hindered by inherent instability under environmental stressors, such as pH fluctuations, heat, oxygen, enzymatic activity, and metal ions [8,15]. Innovative stabilization strategies have emerged to address these challenges, particularly protein-based matrices [10,16] and nano/microencapsulation [17]. Nanoencapsulation technology is widely used in food and pharmaceutical research to protect bioactive compounds from degradation factors, thereby enhancing their bioavailability, bioaccessibility, and functional efficacy [17]. Protein-polyphenol interactions enhance anthocyanin stability while strengthening bioavailability and bioactivity [[8], [9], [10]]. Selecting appropriate natural protein matrices is critical to retaining the integrity and stability of the encapsulated anthocyanin [17,18]. Recent breakthroughs highlighted the synergistic interactions between Lactoferrin and phytochemicals [19,20], unlocking novel applications in nanomedicine and healthcare.

Lactoferrin (LF), an 80 kDa iron-binding glycoprotein within the transferrin family, exhibits versatile biological functions due to its capacity to chelate iron essential for pathogen proliferation and adhere to cellular surfaces via cationic domains. These attributes offer unique advantages as a potent therapeutic agent against microbial infection-driven inflammatory conditions [21]. Abundantly present in mammalian bodily fluids—particularly milk—LF is pivotal in linking innate and adaptive immunity, which is critical for neonatal health [20,22]. Naturally, LF is secreted by inflamed tissues, serving as a frontline defense molecule upon inflammation [23]. This protein stands out for its multifunctional properties, including anti-inflammatory, antimicrobial, antioxidative, and immunoregulatory [22,24]. Despite advances in protein-based encapsulation systems, challenges persist, including structural instability close to its isoelectric point and suboptimal anthocyanin encapsulation efficiency [8]. To effectively utilize anthocyanins, nanotechnology strategies incorporating protein hydrolysis and nano/microencapsulation are essential. To date, bovine lactoferrin (BLF) and ovotransferrin (OTF)—structurally akin to human LF—have demonstrated excellent interactions with polyphenols such as curcumin [25], tannic acid [26], theaflavins [27], and epigallocatechin-3-gallate (EGCG) [28]. Recent research further revealed C3G's binding potential with BLF and OTF [11]. While these works primarily focused on the binding dynamics and their functional impacts on antioxidant capacity and foam stability, investigating lactoferrin-cyanidin-3-glucoside-encapsulated nanoparticles (LF-C3GNPs) could unlock novel therapeutic avenues for managing inflammation and oxidative stress, offering crucial insights for nanomedicine. Given their combined attributes, LF-nanoencapsulation with C3G offers unique, outstanding synergistic benefits that could enhance molecular defense mechanisms against oxidative stress-driven inflammation.

Sestrin2 (*Sesn2*), a stress-responsive protein, is upregulated under oxidative stress to mitigate excessive ROS generation [29,30]. Nuclear factor erythroid-2-related factor-2 (*Nrf2*), a key transcriptional regulator of cellular redox balance, maintains oxidative homeostasis by activating antioxidant genes [31]. Under basal conditions, *Nrf2* remains sequestered in the cytoplasm via binding to Kelch-like ECH-associated protein 1 (Keap1) [32]. During oxidative stress, *Sesn2* induction disrupts the *Keap1*-*Nrf2* interaction, enabling *Nrf2* nuclear translocation and subsequent activation of antioxidant genes, including *Sesn2*, thus heightening the scavenging of ROS [33]. This reversible activation cycle between *Sesn2* and *Nrf2* creates a self-reinforcing antioxidant loop, enhancing cellular resilience. The *Sesn2/Nrf2* signaling uniquely integrates antioxidant, anti-inflammatory, and cytoprotective mechanisms, positioning it as a strategic therapeutic target for restoring homeostasis without disrupting essential physiological functions. Previously, research showed C3G's potential to suppress LPS-induced inflammation by modulating redox and inflammatory mediators, thereby increasing Nrf2 expression [34]. While Nrf2's protective role in mastitis has gained attention [34,35], the mechanism that activates *Sesn2/Nrf2* signaling remains underexplored in mastitis. Thus, we hypothesized that LF-C3GNPs enhance *Sesn2/Nrf2* signaling in the primary defense mammary epithelial cells, preventing inflammation and oxidative damage.

**Scheme 1:**
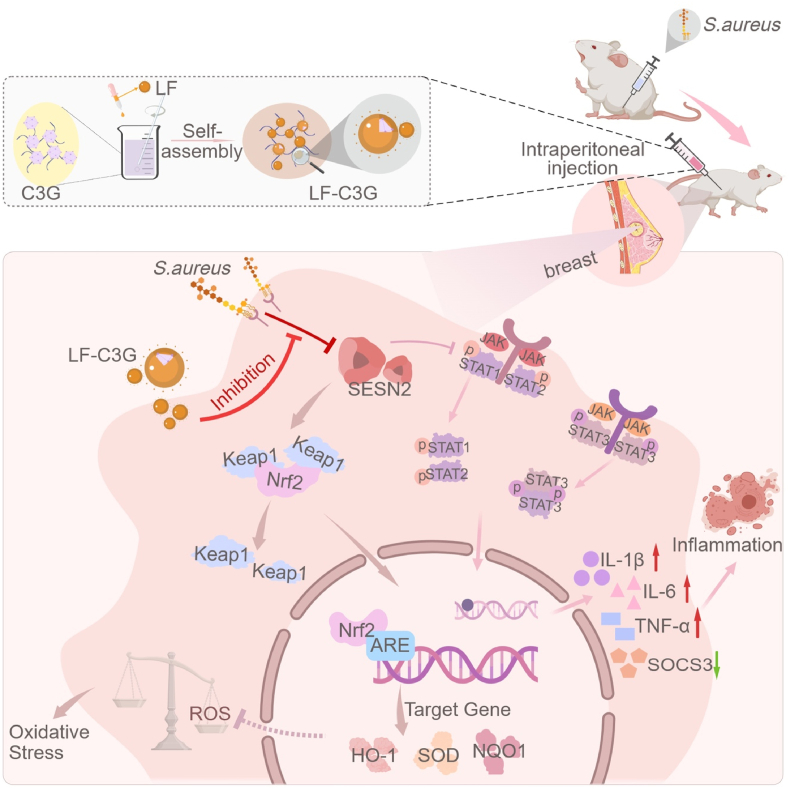
Schematic illustration of lactoferrin-cyanidin-3-glucoside-encapsulated nanoparticles alleviating LTA-induced inflammation and oxidative stress via *Sesn2/Nrf2* activation in mastitis.

In this study, LF-C3G*-*encapsulated nanoparticles (LF-C3GNPs) were engineered and thoroughly characterized to assess their therapeutic potential against LTA-induced inflammation and oxidative stress in mastitis, primarily via modulation of the *Sesn2/Nrf2* signaling pathway (Scheme 1). Experimental models, including *in vivo* murine systems and *in vitro* mammary epithelial cell (HC11) cultures, were employed to assess the effect of LF-C3GNP on inflammatory responses and antioxidant pathways. *In vitro*, LF-C3GNPs counteracted LTA-induced inflammation and hydrogen peroxide (H_2_O_2_)-mediated oxidative damage by restoring redox homeostasis via upregulation of *Sesn2/Nrf2* signaling. Thorough *in vivo* evaluation, including hemocompatibility, H&E staining, and blood biochemistry analysis, confirmed its biocompatibility and therapeutic safety. By bridging the gap of protein-anthocyanin nanoencapsulation, this work advances innovative nanomedicine strategies for managing oxidative stress-driven inflammatory diseases, with potential clinical applications for mastitis.

## 2 Experimental section

### 2.1 Reagents, cells, and bacteria

Cyanidin-3-O-glucoside (C137683*)*, Lactoferrin (L302916), and Dexamethasone (DEX, D137736) were obtained from Aladdin (Shanghai, China). The SOD/MDA/GSH/CAT/MPO (BC5165/BC0025/BC1175/BC0205/BC5715) determination kits were purchased from Solarbio Technology (Beijing, China). Antibodies IL-1β (16806-1-AP), STAT2 (16674-1-AP), SLC7A11 (26864-1-AP), GAPDH (60004-1-Ig), Nrf2 (16396-1-AP), HO-1 (10701-1-AP), Keap1 (10503-2-AP), NQO1 (11451-1-AP), MPO (22225-1-AP), SOCS3 (14025-1-AP), and SESN2 (66297-1-Ig) were obtained from Proteintech BioTechnology (Wuhan, China). Antibodies IL-6 (TD6087), TNF-α (PY19810), STAT1 (T55227), p-STAT1 (TP56498), p-STAT2 (TA8127), STAT3 (T55292), and p-STAT3 (T56566) were obtained from Abmart BioTechnology (Shanghai, China). Antibody GPX4 (ET1706-45) was sourced from HUABIO (Hangzhou, China). Lipoteichoic acid (LTA, L2515), isolated from *Staphylococcus aureus*, was also acquired from Sigma-Aldrich (St. Louis, MO, USA).

The HC11 mammary epithelial cell line (HC11, CL-0616) was sourced from Procell Life Science & Technology Co., Ltd. (Wuhan, China). These cells were maintained in Dulbecco's Modified Eagle Medium (DMEM) containing 10 % fetal bovine serum (FBS) and 1 % penicillin-streptomycin antibiotic mixture. Cultures were incubated under controlled conditions (37 °C, 5 % CO_2_).

### 2.2 Preparation and synthesis of LF-C3GNPs

Lactoferrin (LF) was suspended in phosphate-buffered saline (PBS, pH 7.4) to formulate a 100 μM stock solution. The LF suspension was stored at 4 °C overnight to ensure complete hydration. Cyanidin-3-glucoside (C3G) was dissolved in a citric acid-sodium citrate buffer system (pH 3.0). Adjustments to the pH of both solutions were performed using 10 mM hydrochloric acid (HCl) or sodium hydroxide (NaOH), as previously described [11].

LF-C3GNPs were synthesized by leveraging electrostatic attraction between LF, which carries a positive charge, and C3G, which is negatively charged [36,37]. The LF solution was incrementally introduced into the C3G solution under constant stirring (400 rpm, magnetic stirrer) at a 1:1 mass ratio, adjusted as needed for specific experimental conditions. The combined solution was stirred for 30 min to ensure nanoparticle (NPs) stability and formation. The nanoparticles were then isolated by centrifugation (15,000 rpm, 30 min, 4 °C), after which the LF-C3GNPs pelleted were harvested and stored under optimal conditions for subsequent analytical and experimental applications.

### 2.3 Characterization of LF-C3G nanoencapsulation

The structural and morphological attributes of the nanoencapsulated particles were explored via scanning electron microscopy (SEM; ZEISS-SIGMA HD) and transmission electron microscopy (TEM; JEM-F200). Particle size distribution was determined using dynamic light scattering (DLS; Malvern Zetasizer Nano ZS, UK), while surface charge and colloidal stability were evaluated through **ζ-**potential measurements on the system. Optical properties of the LF-C3GNPs were investigated using ultraviolet–visible (UV–Vis) spectroscopy (Shimadzu UV-2600, Japan). Fourier-transform infrared spectroscopy (FT-IR) using a Nicolet 6700 (Thermo Scientific, USA) confirmed successful nanoparticle synthesis by characterizing chemical interactions between LF and C3G.

### 2.4 Stability and release investigation

The structural integrity of LF-C3GNPs was harvested across multiple solvents—including deionized water (H_2_O), Dulbecco's Modified Eagle Medium (DMEM), fetal bovine serum (FBS), and 0.9 % sodium chloride (NaCl)—using dynamic light scattering (DLS) analysis at predetermined intervals (0, 2, 4, 6, 8, 10, and 12 h). Digital images of the encapsulated NPs suspensions were captured to track aggregation or sedimentation over time.

The release kinetics of C3G from free C3G and LF-C3GNPs were evaluated using ultraviolet–visible (UV–Vis) spectroscopy. Samples were incubated under mild agitation at 37 °C, with aliquots withdrawn at predetermined intervals (0, 1, 2, 4, 6, 8, 10, 12, 16, 20, 24, 36, and 48 h). Absorbance measurements were performed on these aliquots to quantify cumulative C3G release over time via spectral analysis. Additionally, LF-C3GNPs were subjected to 1 mM hydrogen peroxide (H_2_O_2_) for oxidative stress investigation. Alterations in nanoencapsulated particle dimensions pre- and post-exposure were quantified via DLS. The H_2_O_2_ scavenging efficacy of free C3G and LF-C3G nanoencapsulation was further compared using UV–Vis spectrophotometric analysis.

### 2.5 Cell viability and cytotoxicity assays

To evaluate the viability of HC11 mammary epithelial cells following treatment, the Cell Counting Kit-8 (CCK-8) assay (Biosharp, Hefei, China) was employed. The cells were grown in Dulbecco's Modified Eagle Medium (DMEM) containing 10 % fetal bovine serum (FBS) and 1 % penicillin-streptomycin antibiotics under standard incubation conditions (37 °C, 5 % CO_2_). For the assay, cells were plated in 96-well plates at a density of 5 × 10^3^ cells per well and allowed to adhere overnight. After adherence, the cells were treated with graded concentrations of free C3G, LF, and LF-C3GNPs for 24 h. After treatment, cell viability was determined by replacing the medium with 10 % CCK-8 solution, incubating for 1 h, and analyzing absorbance at 450 nm using a Bio-Rad microplate reader (Hercules, CA, USA).

$$\text{Cell}\;\text{viability}\;\left(\%\right)=\left(\frac{{Α}_{\exp}}{{Α}_{ctrl}}\right)\;x\;100$$

*A*_exp_ = Absorbance of experimental group
*A*_ctrl_ = Absorbance of a control group

### 2.6 Cell culture and treatment experiments for inflammation

HC11 mammary epithelial cells were maintained in DMEM supplemented with 10 % FBS and 1 % penicillin-streptomycin under standard incubation conditions (37 °C, 5 % CO_2_), following established protocols [38]. Cells were seeded into 6-well plates at a density of 10^6^ cells/mL and allocated into six experimental groups (n = 6): control (FBS-free medium), LTA Group (Exposed to 5 μg/mL LTA for 24 h), C3G + LTA (Pre-incubated with 100 μg/mL C3G for 2 h, followed by 5 μg/mL LTA for 24 h), LF + LTA (Pre-treated with 100 μg/mL LF for 2 h, then 5 μg/mL LTA for 24 h), LF-C3G + LTA (Pre-treated with 100 μg/mL LF-C3G nanoparticles for 2 h, then 5 μg/mL LTA for 24 h), DEX + LTA (Pre-incubated with 100 μg/mL DEX for 2 h, followed by 5 μg/mL LTA for 24 h).

### 2.7 ROS detection using DCFH-DA staining

Intracellular ROS levels were measured using the fluorescent probe 2′,7′-dichlorofluorescin diacetate (DCFH-DA) from a commercial ROS detection kit (BL714A, Biosharp). Cells were seeded into 12-well plates and cultured for 24 h before treatments with C3G (100 μg/mL), LF (100 μg/mL), or LF-C3GNPs (100 μg/mL) for 2 h. Subsequently, cells were exposed to 400 mM H_2_O_2_ for 24 h to stimulate ROS production. Afterward, cells were rinsed three times using serum-free medium and treated with DCFH-DA (1:1000 dilution, 10 μM) under incubation at 37 °C for 30 min. The unbound probe was removed via three additional washes with PBS. Intracellular ROS fluorescence was visualized using a fluorescence microscope, and quantitative intensity analysis was conducted using ImageJ software (National Institutes of Health, USA).

### 2.8 Mitochondrial membrane potential assay

The mitochondrial membrane potential (ΔΨm) was evaluated using the fluorescent dye JC-1, a standard tool for this measurement [39]. The JC-1 assay kit (Solarbio, Cat# M8650) was applied according to the manufacturer's protocol. Cells were grown in 12-well plates for 24 h and then treated with C3G (100 μg/mL), LF (100 μg/mL), or LF-C3GNPs (100 μg/mL) for 2 h. Subsequently, cells were exposed to 400 mM H_2_O_2_ for 24 h. Following two rinses with PBS, cells were incubated in JC-1 staining solution at 37 °C for 30 min. Subsequently, two additional PBS washes were performed. Fluorescence microscopy was used to image the stained cells, and quantitative analysis of fluorescence intensity was performed using ImageJ software.

### 2.9 C11-BODIPY assay

The fluorescent probe C11-BODIPY, recognized for its dependability in assessing lipid peroxidation and antioxidant activity within membrane models and living cells, was employed as outlined in previous studies [40]. HC11 cells were analyzed for lipid peroxidation using C11-BODIPY (Sigma-Aldrich, USA). Following a 24-h incubation period, cells were treated with C3G (100 μg/mL), LF (100 μg/mL), or LF-C3GNPs (100 μg/mL) for 2 h, and subsequently, cells were exposed to 400 mM H_2_O_2_ for 24 h. After which, the plates were rinsed twice with PBS. Cells were then incubated for 30 min in DMEM containing 5 μM C11-BODIPY at 37 °C and 5 % CO_2_. After staining, two additional PBS washes were performed to remove residual dye. Fluorescence microscopy images were acquired and analyzed quantitatively using ImageJ software [41].

### 2.10 Evaluation of the antioxidant activity of LF-C3GNPs

Proteins were extracted from treated HC11 cells using RIPA lysis buffer, and the total protein content was measured via a bicinchoninic acid (BCA) assay according to the manufacturer's instructions. Key oxidative stress biomarkers, such as catalase (CAT), superoxide dismutase (SOD), malondialdehyde (MDA), and glutathione peroxidase (GPx), were evaluated in the cell lysate supernatant. Enzymatic activities of the markers were assessed and quantified using standardized commercial assay kits, following the manufacturers' protocols.

### 2.11 Animal treatment assay

Animal investigations were conducted following the National Institutes of Health (NIH) guidelines and approved by the Animal Ethics Committee of Jiangsu Normal University, China [Protocol No. JSNU-IACUC-2025027]. Female and male ICR mice (6–8 weeks old, 20–30 g) were acquired from the Xuzhou Medical University Animal Experiment Center. Mice were housed for one week under controlled conditions (22 °C, 12-h light/dark cycle) with ad libitum access to food and water before the experiments.

Here, we explore the therapeutic effect of LF-C3GNPs on *S. aureus*-induced inflammation in a pregnant mouse mastitis model. The female mice were randomly allocated into six groups: control administered PBS, infection model (inoculated with *S. aureus*, 1 × 10^8^ CFU), C3G treatment (50 mg/kg) + *S. aureus* (1 × 10^8^ CFU), LF treatment (50 mg/kg) + *S. aureus* (1 × 10^8^ CFU), LF-C3GNPs treatment (50 mg/kg) + *S. aureus* (1 × 10^8^ CFU) and DEX (100 mg/kg) + *S. aureus* (1 × 10^8^ CFU). C3G, LF, LF-C3GNPs, and *S. aureus* + 50 mg/kg positive Control dexamethasone (DEX) selected for its well-documented anti-inflammatory properties [42,43]. These treatments were administered via intraperitoneal injection 1 h before *S. aureus* challenge. The mastitis model was established according to previously described protocols [42], and disease progression was monitored by histological analysis of mammary tissue and quantification of inflammatory cytokines.

Pregnant mice (5–7 days postpartum) were isolated from their offspring for 3 h, anesthetized, and the mammary gland region (L4/R4) disinfected with 75 % ethanol. *S. aureus* was injected directly into the mammary gland ducts, while the control group received PBS. Twenty-four hours post-infection, mice were euthanized via cervical dislocation. Mammary tissues were excised, photographed for macroscopic assessment, and stored at −80 °C for subsequent analyses.

### 2.12 Histological analysis and immunofluorescence staining of mammary tissue

In the murine model of *S. aureus* infection, mammary gland tissues from mice were fixed in 4 % paraformaldehyde, gradually dried through a dehydration series, and embedded in paraffin wax. Thin sections (4 μm thickness) were sliced using a rotary microtome, followed by dewaxing, hydration, and staining with hematoxylin and eosin (H&E). Morphological alterations in mammary tissue were analyzed using a light microscope (Olympus, Tokyo, Japan). Similar histological evaluations of vital organs, including the heart, liver, spleen, lungs, and kidneys, were performed to evaluate systemic biocompatibility using the standardized tissue processing and staining protocols.

For tissue immunofluorescence staining, mouse mammary tissue sections embedded in paraffin were first deparaffinized and subjected to antigen retrieval. Following this, sections were blocked using goat serum to minimize non-specific binding and incubated overnight at 4 °C with a rabbit-derived primary antibody specific for SESN2 (Proteintech, 66297-1-Ig), Nrf2 (Proteintech, 16396-1-AP), and TNF-α (Abmart, PY19810) at a 1:300 dilution. Subsequently, sections were treated with a fluorescein isothiocyanate (FITC)-conjugated donkey anti-rabbit secondary antibody diluted 1:200 for 1 h at room temperature. Nuclei were counterstained with DAPI to enable cellular localization. Fluorescence imaging was performed using a microscope to capture high-resolution images of the labeled tissues.

### 2.13 Bacterial survival assessment in mammary tissues

Tissue samples from different groups were harvested following LF, C3G, and LF-C3GNPs treatments, homogenized in PBS, diluted, and plated on agar to assess bacterial growth at 37 °C for 24 h; the colonies were subsequently quantified.

### 2.14 Western blot analysis

Total proteins were extracted from mammary tissue samples by homogenizing them in RIPA buffer containing a phosphatase inhibitor. The homogenate was centrifuged at 12,000 g for 15 min at 4 °C to isolate the supernatant. Protein concentrations were analyzed using the BCA Protein Assay Kit (Thermo Fisher Scientific). The proteins were then separated via electrophoresis on a 10 % sodium dodecyl sulfate-polyacrylamide gel (SDS-PAGE) and blotted onto nitrocellulose membranes for further analysis. Subsequently, after blocking with 5 % non-fat milk in Tris-buffered saline (TBS) for 2 h, membranes were incubated overnight at 4 °C with primary antibodies diluted 1:1000 in TBST, including IL-1β, IL-6, TNF-α, MPO, SOCS3, STAT1, p-STAT1, STAT2, p-STAT2, STAT3, p-STAT3, GPX4, SLC7A11, Keap1, Nrf2, SESN2, HO-1, NQO1 and GAPDH.

After washing, membranes were incubated for 2 h at room temperature with HRP-conjugated secondary antibodies (1:1000 dilution; Abcam, USA). Protein bands were visualized using an enhanced chemiluminescence substrate (Biosharp, China) and imaged with a chemiluminescence detection system (Tanon, China). Band intensities were quantified using ImageJ software (NIH).

### 2.15 Quantitative real-time PCR (qRT-PCR)

RNA was isolated from cell cultures and mammary tissue samples using the RNeasy extraction kit (Qiagen, Germany). Complementary DNA (cDNA) was synthesized from the extracted RNA using a reverse transcription kit (TaKaRa, Japan). Quantitative real-time PCR (qRT-PCR) was performed with SYBR Premix Ex Taq™ II (TaKaRa, Japan) to amplify the target genes, including IL-1β, IL-6, TNF-α, MPO, and STAT family members. Gene expression levels were normalized to the reference gene GAPDH and calculated using the 2^−ΔΔCt^ method, with results presented as fold-change values relative to untreated controls. Primer sequences for all analyzed genes are detailed in Table S1.

### 2.16 Immunofluorescence analysis

HC11 mammary epithelial cells were maintained in culture for 24 h and subsequently exposed to C3G (100 μg/ml), LF (100 μg/ml), LF-C3GNPs (100 μg/ml), or LTA (5 μg/ml). Following treatment, cells were fixed with 4 % paraformaldehyde for 30 min and incubated in a 5 % bovine serum albumin (BSA) solution for 30 min to minimize nonspecific binding. After three rinses with PBS, samples were treated with primary antibodies (1:200 dilution) specific to SESN2, Nrf2, and TNF-α at 37 °C for 2 h. After additional PBS washes, cells were incubated with a Fluor488-conjugated goat anti-rabbit IgG secondary antibody at 37 °C for 30 min. Nuclei were labeled with DAPI, and fluorescent signals were captured using a fluorescence microscope (Olympus, Japan). Signal intensity quantification was performed using ImageJ software (NIH, USA).

### 2.17 Safety evaluation of LF-C3G NPs

#### 2.17.1 Hemolysis activity assay

Hemolysis was evaluated following an adapted methodology from previous research [44]. Mouse red blood cells were harvested by centrifuging 0.5 mL of whole blood at 3500 rpm for 5 min. The cells were then rinsed three times with sodium thiobarbital buffer and used to prepare a 5 % erythrocyte suspension (v/v). Incremental concentrations of LF-C3GNPs were mixed with the erythrocyte preparation and gently agitated (100 rpm) at 37 °C for 1 h. After incubation, samples were centrifuged at 3500 rpm for 5 min to separate intact red blood cells from the supernatant. Spectrophotometry at 545 nm was used to measure the optical density (OD) of the supernatant, quantifying hemoglobin release as an indicator of hemolysis. PBS was used as a negative control, providing an isotonic environment to prevent hemolysis. Owing to its hypotonic condition, deionized water served as a positive control, inducing maximum hemolysis. The hemolysis percentage was calculated as follows:

$$\text{Hemolysis}\;\text{ratio}\;\left(\%\right)=\left(\frac{{\text{OD}}_{\text{sample}}\;‐\;{\text{OD}}_{\text{PBS}}}{{\text{OD}}_{\text{water}}\;‐\;{\text{OD}}_{\text{PBS}}}\right)\times 100\%\text{.}$$

OD_sample_ represents the absorbance of erythrocytes exposed to different concentrations of LF-C3GNPs, respectively.

#### 2.17.2 Calcein-AM/PI live-dead cell staining

HC11 cells were seeded at a density of 2 × 10 ^4^ cells/well in 24-well plates to adhere overnight. They were then incubated with various concentrations of LF-C3GNPs for 24 h. Subsequently, cell monolayers were washed twice with PBS, stained with Calcein-AM and propidium iodide (PI) according to the manufacturer's protocol, and incubated for 15 min at 37 °C. Fluorescence signals were then acquired using fluorescence microscopy with appropriate filters. The ratio of Calcein-AM-stained live cells to PI-stained dead cells was measured using ImageJ software to assess cell viability and cytotoxicity.

#### 2.17.3 Blood biochemistry assessment

To evaluate the systemic safety profile of LF-C3G NPs, serological tests were conducted to monitor liver and kidney function markers. Mice were injected with a dose of 50 mg/kg LF-C3GNPs, and blood samples were subsequently harvested from the treated mice. The blood samples were centrifuged at 3000 rpm for 10 min at 4 °C to obtain serum. This serum was then analyzed for markers of liver and kidney function, including alanine aminotransferase (ALT), aspartate aminotransferase (AST), blood urea nitrogen (BUN), and creatinine (CRE). These biomarkers were analyzed using standardized commercial assay kits (Nanjing Jiancheng, China) following the manufacturer's guidelines.

#### 2.17.4 Long-term biosafety investigation *in vivo*

Mice received IV injections of LF-C3GNPs (50 mg/kg) or PBS for 14 days. Serum biomarkers (ALT, AST, BUN, CRE) and hematological parameters were analyzed at days 7 and 14. Organs were harvested for H&E staining.

### 2.18 Statistical analysis

Data were analyzed using SPSS software (v21.0, IBM, USA) and reported as mean ± standard deviation (SD). Graphical data representations were conducted using GraphPad Prism 8.3 (San Diego, CA, USA. Group comparisons were analyzed via one-way ANOVA, followed by LSD post hoc testing, with a statistical significance threshold of *p < 0.05*.

## 3 Results and discussion

### 3.1 Synthesis and characterization of LF-C3G-encapsulated nanoparticles

**Fig. 1:**
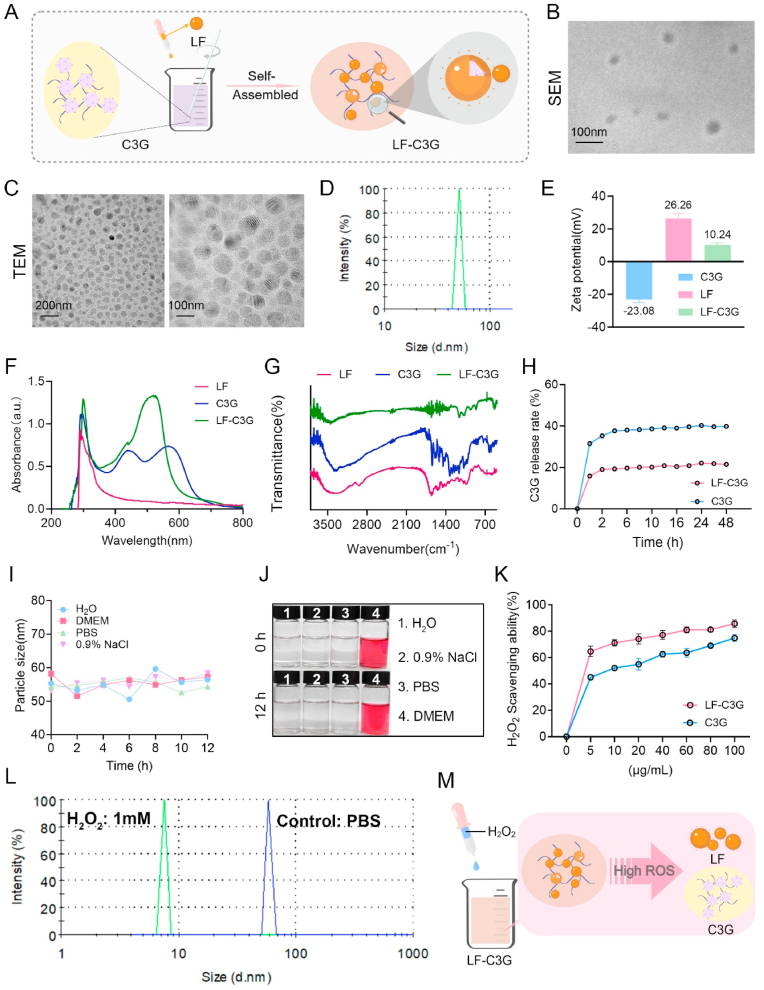
Synthesis and characterization of LF-C3G-encapsulated nanoparticles. **(A)** Schematic illustration of the synthesis of LF-C3G-encapsulated nanoparticles (LF-C3GNPs). **(B)** SEM images of LF-C3GNPs (scale bar = 100 nm). **(C)** TEM images (scale bar = 100 nm, 200 nm). **(D)** Particle size distributions of LF-C3GNPs via DLS. **(E)** Zeta potential values to assess surface charge and colloidal stability of LF-C3GNPs. **(F)** UV–visible absorbance spectra of LF-C3GNPs. **(G)** FTIR spectrum of LF-C3GNPs. **(H)** The controlled release profiles of free C3G and LF-C3GNPs via UV–Vis spectroscopy. **(I)** Colloidal stability of LF-C3GNPs in diverse media (deionized water, DMEM, PBS, 0.9 % NaCl) by tracking hydrodynamic diameter changes over time via DLS. **(J)** Visual inspection and photographic image of LF-C3GNPs in different solutions. **(K)** Quantitative analysis of the scavenging rate of H_2_O_2_ by LF-C3GNPs relative to free C3G. **(L)** Changes in the size of LF-C3GNPs induced by ROS (H_2_O_2_) stimulation, as determined by DLS. **(M)** Schematic illustration of the H_2_O_2_-responsive mechanism of LF-C3GNPs.

LF is nanoencapsulated within C3G using electrohydrodynamic nanotechnology, leveraging electrostatic attraction between LF, which carries a positive charge, and C3G, which is negatively charged at pH 3 to create LF-C3G-encapsulated nanoparticles (LF-C3GNPs) (Fig. 1A). This nanotechnology, recently highlighted for its efficiency and scalability [36,37], offers a cost-effective and adaptable approach for encapsulating bioactive compounds, such as C3G. Its precision is especially beneficial for preserving thermally and photolabile molecules such as anthocyanins.

SEM and TEM imaging revealed LF-C3GNPs with spherical uniformity, smooth surfaces, and an average diameter of ∼50 nm (Fig. 1B and C). Spherical nanoparticles are often preferred for drug delivery owing to their uniform cellular uptake and reduced immune recognition. The average diameter of ∼50 nm places LF-C3GNPs in an optimal size range for systemic circulation and tissue penetration. Nanoparticles <100 nm can exploit the enhanced permeability and retention effect in inflamed tissues, enhancing targeted delivery. DLS measurements showed a hydrodynamic size of ∼50 nm in phosphate buffer solution (pH 7.4) (Fig. 1D), aligning with criteria for NPs stability, where smaller sizes and optimal ζ-potential values (≥±30 mV) minimize aggregation [45]. This consistency suggests colloidal stability, essential for maintaining NPs’ integrity in biological fluids. Smaller nanoparticles (<100 nm) also evade rapid renal clearance, prolonging circulation time and improving therapeutic availability. The ζ-potential of LF-C3GNPs (10.24 mV), relative to LF (26.26 mV) and C3G (−23.08 mV), confirmed successful nanoencapsulation (Fig. 1E). The strong positive charge of LF facilitated rapid electrostatic binding with C3G, reducing hydrophilic loss and enhancing dispersion stability via surface charge modulation [25].

UV–vis spectra are commonly utilized to explore alterations in structural and intermolecular interactions [46]. UV–vis spectroscopy detected the absorbance characteristics of LF's aromatic residue peak at 280 nm and C3G's anthocyanin chromophore peak at 453 nm (Fig. 1F). C3G exhibits pH-dependent behaviors, with the flavylium cation showing dominant absorbance at pH 3, which is associated with its anthocyanin chromophore [11]. The LF-C3GNPs spectrum retained both peaks, 280 nm and 453 nm (Fig. 1F), indicating the successful dual-component integration. FTIR was conducted to identify the functional groups involved in the interactions of LF-C3GNPs. FTIR-recorded the spectra showed that LF exhibited rich phenolic hydroxyl stretching vibrations at amide I (C=O, ∼1650 cm^−1^) and amide II (N–H, ∼1540 cm^−1^) bands, while C3G showed O–H (∼3300 cm^−1^) and aromatic C=C (∼1600 cm^−1^) stretches (Fig. 1G). Fourier-transform infrared (FTIR) spectroscopy analysis revealed that lactoferrin (LF) and cyanidin-3-glucoside (C3G) both exhibited C=C stretching vibrations within their aromatic ring systems, observed in the spectral range of 1500–1600 cm^−1^. The FTIR profile of LF-C3GNPs retained the distinctive absorption bands of LF and C3G while further displaying additional peaks corresponding to carboxylic acid (COOH) stretching vibrations between 1600 and 1700 cm^−1^, likely shifted in amide peaks and hydroxyl group positions, and altered intensities suggested hydrogen bonding and π–π stacking, indicative of non-covalent interactions that stabilize the complex. Overall, these findings indicate that protein and polyphenols interact through non-covalent (hydrophobic, ionic, and hydrogen bonding) or covalent bonds [47], with non-covalent forces primarily driving stability and biofunctionality of LF-C3GNPs.

### 3.2 *In vitro* controlled release and ROS-responsive degradation of LF-C3GNPs

To simulate the drug release rate of LF-C3GNPs, 1 mL of C3G and LF-C3GNPs solutions underwent dialysis against 10 mL of a 20 % ethanol-PBS dissolution buffer (pH 7.4) under controlled conditions at 37 °C. As expected, LF-C3GNPs exhibited C3G release occurring gradually relative to free C3G (Fig. 1H). This controlled release mechanism, likely due to nanoencapsulation, prolongs antioxidant efficacy in physiological environments and ensures the bioavailability. This suggests that LF-C3GNPs present a stable and sustained delivery system for C3G in nanomedicine applications. Colloidal stability assays in diverse media (H_2_O, DMEM, FBS, 0.9 % NaCl) revealed consistent particle size in water and saline over 12 h (Fig. 1I and J). Moderate size increases in DMEM/FBS were attributed to protein adsorption, though no significant aggregation occurred. Visual inspection and photographic documentation confirmed these findings (Fig. 1J), with turbidity observed only in nutrient-rich media. These features spot LF-C3GNPs as a promising candidate for drug delivery systems.

Given the free radical scavenging capabilities of C3G and its propensity to undergo structural changes and break down into smaller molecules when exposed to H_2_O_2_ [48], we assessed LF-C3GNPs’ capability to scavenge H_2_O_2_ and their structural integrity under oxidative conditions. As expected, free C3G and LF-C3GNPs demonstrated dose-dependent H_2_O_2_ scavenging, with LF-C3GNPs exhibiting superior and prolonged activity at lower drug concentrations (Fig. 1K). These findings suggest that nanoencapsulating LF within C3G stabilizes the latter against degradation, enhancing drug retention and enabling sustained antioxidant release [11,12]. Finally, we assessed the size of LF-C3GNPs after exposure to 1 mM H_2_O_2_ for 10 min to investigate ROS-responsiveness under oxidative stress conditions.

DLS analysis showed reduced LF-C3GNPs size (Fig. 1L), indicating that NPs were disrupted or disintegrated in response to ROS. Unlike conventional LF-curcumin or β-lactoglobulin-C3G complexes [49,50], which rely on passive diffusion, LF-C3GNPs substantially leverage a ROS-triggered release mechanism. This ensures precise delivery of C3G to inflamed mammary tissues, where oxidative stress is elevated, enhancing therapeutic specificity and minimizing off-target effects. Together, LF-C3GNPs effectively mitigate oxidative damage by efficiently scavenging H_2_O_2_ radicals, and it has further potential for targeted antioxidant delivery in ROS-rich microenvironments (Fig. 1M).

### **3.3** Effects of LF-C3GNPs on LTA-induced inflammation in HC11 cells

**Fig. 2:**
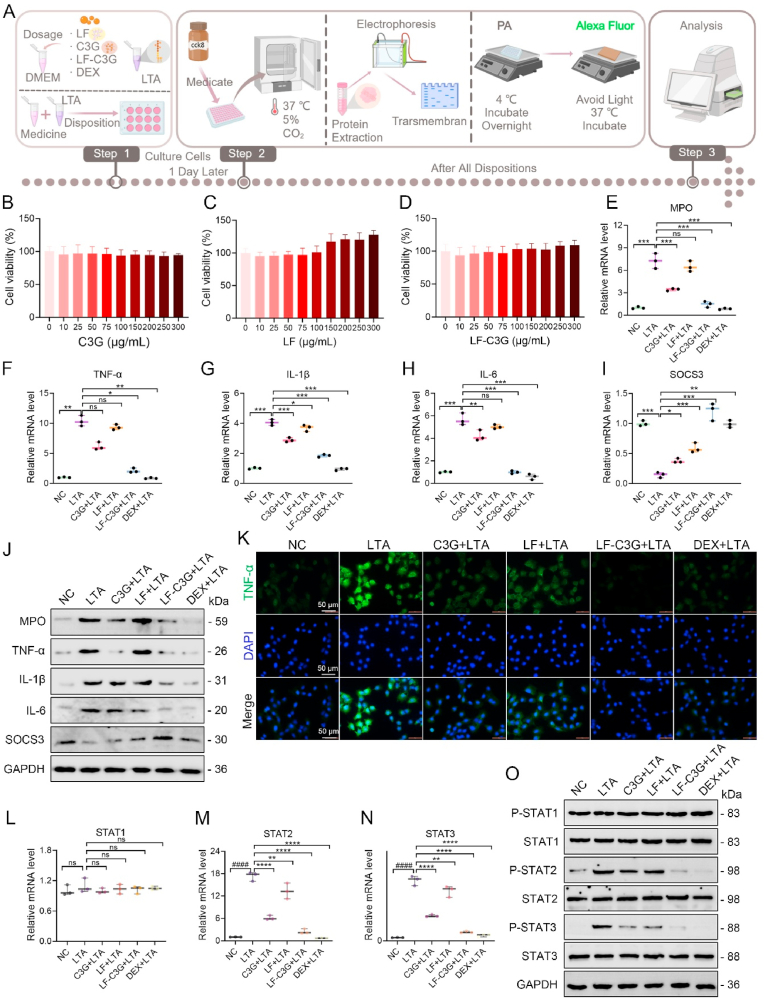
Effects of LF-C3GNPs on LTA-induced inflammation in HC11 cells. **(A)** Schematic diagram of cell culture protocol, LTA-induced inflammation in HC11 pretreated with LF-C3GNPs, the experimental verification. (**B-D)** Cell viability of LF, C3G, and LF-C3GNPs. **(E**–**J)** mRNA **(E**–**I)** and protein **(J)** expression levels of MPO, TNF-α, IL-1β, IL-6, and SOCS3 in HC11 cells. **(K)** Immunofluorescence staining of TNF-α. Scale bar: 50 μm. **(L**–**O)** mRNA **(L**–**N)** and protein **(O)** expression levels of phosphorylated STATs (p-STAT1, p-STAT2, and p-STAT3) and STAT1, STAT2, and STAT3 in HC11 cells after different treatments. The data are presented as mean ± standard deviation (SD) from three independent replicates. Statistical significance is designated as **^#^***p<0.05* compared to the untreated control group; **∗***p<0.05*, **∗∗***p<0.01*, **∗∗∗***p<0.001*, and **∗∗∗∗***p<0.0001* relative to the LTA-treated groups.

Inflammation is regulated via diverse mechanisms. Mammary epithelial cells are the primary defense mechanism against pathogen-induced inflammation in the mammary gland. Dysfunction in these cells impairs the integrity of the blood-milk barrier and recruits immune cells to the site [5]. Notably, exposure to LTA, a significant virulence factor in *S.* *aureus* [51], triggers the release of inflammatory mediators to modulate infection dynamics [52]. Persistent inflammation, however, causes the secretion of cytokines that disrupt the microenvironment, leading to tissue injury and immune dysregulation [3]. Thus, we explored the anti-inflammatory effects of LF-C3GNPs on LTA-induced mastitis to gain insights into inflammatory signaling in HC11 cells (Fig. 2A). The CCK8 assay revealed that concentrations of LF, C3G, and LF-C3GNPs (0–300 μg/mL) had no adverse effect on the viability of HC11 cells (Fig. 2B–D), suggesting their excellent cytocompatibility. A broad concentration range of 10–300 μg/ml was necessary to ensure a complete assessment of biocompatibility for C3G, LF, and LF-C3GNPs, establishing a definitive safety threshold for subsequent experiments. Consistent with our previous research [53] and other studies, similar anthocyanin concentrations (50, 100, and 200 μg/ml) significantly enhanced cell viability under stress conditions [48].

**Fig. 3:**
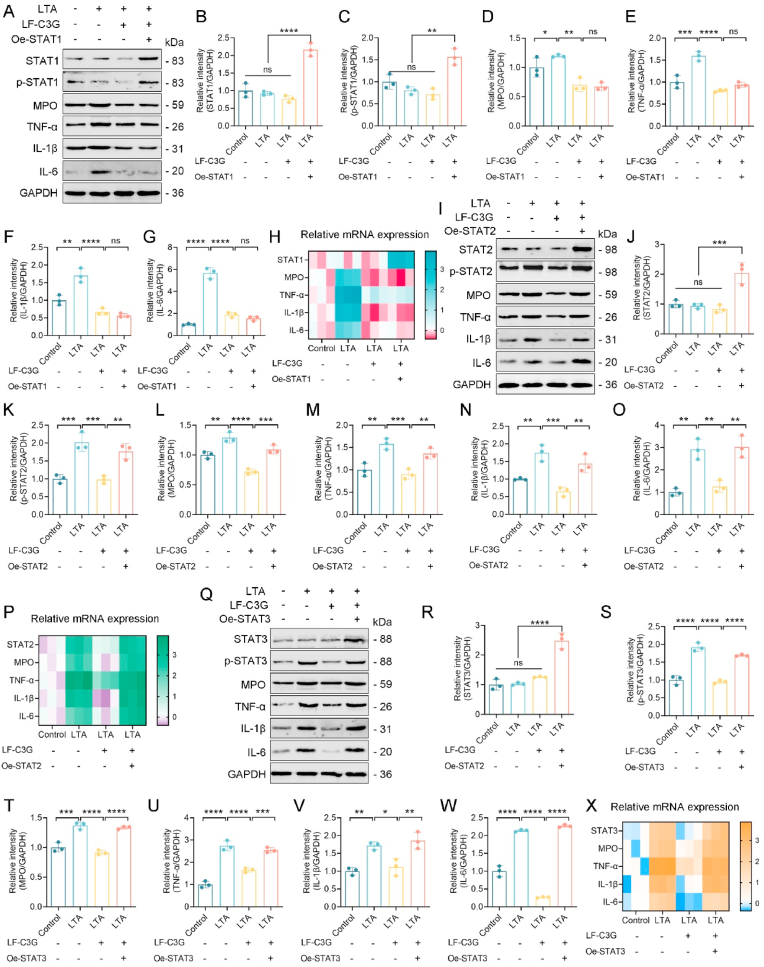
LF-C3GNPs preferentially target STAT2/3 rather than STAT1 in LTA-stimulated cells. **(A**–**G)** Western blot and quantification showing the effects of LF-C3GNPs and STAT1 overexpression (Oe-STAT1) on STAT1/p-STAT1 and downstream inflammatory mediators (MPO, TNF-α, IL-1β, IL-6) in LTA-stimulated cells. **(H)** Heatmap of relative mRNA expression of genes. **(I**–**O)** Western blot and quantification demonstrating the effects of LF-C3GNPs and STAT2 overexpression (Oe-STAT2) on STAT2/p-STAT2 and downstream mediators. **(P)** Heatmap of relative mRNA expression of genes. **(Q**–**W)** Western blot and quantification showing the effects of LF-C3GNPs and STAT3 overexpression (Oe-STAT3) on STAT3/p-STAT3 and downstream mediators. **(X)** Heatmap of relative mRNA expression of genes. Data are presented as mean ± SD (n = 3). *∗p < 0.05, ∗∗p < 0.01, ∗∗∗p < 0.001, ∗∗∗∗p < 0.0001*; ns, not significant.

Myeloperoxidase (MPO), a hallmark enzyme in polymorphonuclear neutrophils and monocytes in mice and humans [54], is a relevant biomarker for neutrophil and macrophage infiltration in inflamed mammary tissues. As expected, LTA exposure increased MPO levels (Fig. 2, Fig. 3J, and Fig. S1A), suggesting heightened neutrophil recruitment, while LF-C3GNPs pretreatment before LTA significantly restored these levels relative to LF or free C3G-treated groups. DEX, as the positive control, showed moderate results relative to LF-C3GNPs. Pro-inflammatory cytokine responses are key factors in initiating the progression of LTA-induced mastitis [42]. We subsequently explored the effects of LF-C3GNPs on pro-inflammatory cytokines in inflamed mammary epithelial cells, focusing on TNF-α, IL-1β, and IL-6, which are documented key markers of inflammation involved in inflammation-related disease such as mastitis. LTA stimulation upregulated mRNA and protein expression levels of IL-1β, IL-6, and TNF-α, an effect significantly attenuated by LF-C3GNPs compared to LF, free C3G, or DEX-treated groups (Fig. 2F–H, 2J, and Fig. S1B–D). These findings align with previously reported research highlighting the central role of MPO, TNF-α, IL-1β, and IL-6 in inflammatory pathologies [55]. Similarly, LTA stimulation downregulated SOCS3 mRNA and protein expression levels, an effect significantly improved by LF-C3GNPs (Fig. 2I and J, and Figure S1E). SOCS proteins, including SOCS3, act via negative feedback to block cytokine signal transduction, which prevents excessive and prolonged inflammation. SOCS3 dysregulation alters milk protein synthesis and heightens mammary epithelial cell susceptibility to *S. aureus* infections [56,57]. TNF-α is often considered the most important cytokine involved in inflammation, mediating inflammatory responses, promoting the activation of other cytokines, initiating the acute phase response, and influencing various immune cells [58]. Consistent with this evidence, immunofluorescence staining of TNF-α in HC11 cells showed a notable decrease in fluorescence intensity following treatment with LF-C3GNPs relative to those observed in LF, free C3G, or DEX-treated groups (Fig. 2K, Fig. S1F), consistent with Western blot and qPCR results. These results imply that the effect of LF-C3GNPs similarly affected the release of pro-inflammatory cytokines and MPO activity in inflamed mammary epithelial cells.

The JAK-STAT signaling plays a crucial role in mammary function, lactation, and immune regulation. In LTA-induced inflammation, however, hyperactivation of this pathway through the phosphorylation of STAT3 and STAT2 triggers the release of pro-inflammatory cytokines [59]. Thus, we explored the effect of LF-C3GNPs on JAK-STAT-mediated inflammation in mammary function. The results showed that LTA exposure significantly triggered phosphorylation of STAT3 and STAT2 (Fig. 2L–N, Fig. 2O, and Fig. S2), a hallmark of inflammatory pathway activation. LF-C3GNPs treatment significantly attenuated LTA-driven phosphorylation without altering STAT1 expression levels relative to LF, free C3G, or DEX-treated groups. This suggests a selective inhibition of pathway activation, as STAT1 is primarily associated with antiviral defense rather than antibacterial immunity [60]. In addition, STAT1 is predominantly activated by interferons (IFNs) via JAK1/TYK2 kinases, whereas STAT2 and STAT3 are downstream of cytokine receptors, such as IL-6R, signaling through JAK1/JAK2 [61]. LF-C3GNPs may preferentially target receptors or upstream JAK2 kinases associated with inflammatory cytokines, such as IL-6, rather than IFN-driven STAT1 activation. STAT3 activation is redox-sensitive, requiring ROS for sustained phosphorylation. As a potent antioxidant, LF-C3GNPs likely suppress ROS-mediated activation of STAT3/STAT2, while STAT1 activation is less ROS-dependent, potentially explaining its resistance to LF-C3GNPs.

Inflammatory cytokine release, including IL-1β, IL-6, and TNF-α, is driven by the JAK-STAT signaling pathway, which is activated when STAT2 and STAT3 become phosphorylated. Our findings demonstrate that LF-C3GNPs reduced this release in mammary epithelial cells by modulating JAK-STAT phosphorylation. Since this pathway is essential for normal mammary gland function but also linked to disorders like mastitis when dysregulated [62,63], controlling its activity is crucial. While LTA-induced mastitis causes sustained inflammatory signaling, the treatment with LF-C3GNPs successfully restored homeostasis, thereby alleviating the cytokine storms. These findings demonstrate that the anti-inflammatory effects of LF-C3GNPs in HC11 cells are likely mediated through the modulation of JAK-STAT2/3 signaling mechanisms, which play a prominent role in cytokine-mediated inflammatory responses. By targeting a pathway central to both mammary physiology and pathology, LF-C3GNPs address inflammation without disrupting normal gland function, preserving milk synthesis and lactation capacity.

### 3.4 LF-C3GNPs preferentially target STAT2/3 in LTA-stimulated cells

To elucidate the mechanistic basis for the preferential regulation of STAT2/3 signaling by LF-C3GNPs, we utilized an overexpression investigation of STAT1, STAT2, and STAT3. The results showed that ectopic expression of STAT1 (Oe-STAT1) markedly enhanced STAT1 and p-STAT1 levels. Upon LTA stimulation, inflammatory mediator expression, including MPO, TNF-α, IL-1β, and IL-6 were promoted. However, the addition of LF-C3GNPs still attenuated these pro-inflammatory responses, and no significant differences were observed between the LTA + LF-C3GNPs and LTA + LF-C3GNPs + Oe-STAT1 groups. These findings indicate that LF-C3GNPs exert only a limited modulatory effect on STAT1 activation, with little impact on STAT1-associated inflammatory gene expression (Fig. 3A–H). In contrast, overexpression of STAT2 (Oe-STAT2) or STAT3 (Oe-STAT3) led to striking changes. LTA challenge induced robust phosphorylation of STAT2/3 (p-STAT2, p-STAT3) and significantly increased inflammatory mediator levels. Importantly, LF-C3GNPs treatment strongly inhibited this activation, as evidenced by the decreased expression of MPO, TNF-α, IL-1β, and IL-6 in the LTA + LF-C3GNPs groups. When STAT2 or STAT3 were ectopically expressed, this suppressive effect of LF-C3GNPs was largely abolished, restoring inflammatory mediator expression to high levels (Fig. 3I–X). The potent inhibition of this well-known master regulator of inflammation underscores the therapeutic promise of LF-C3GNPs. However, the partial inhibition of the potent Oe-STAT1 response implies that LF-C3GNPs act upstream of the STATs themselves, possibly on common kinases like JAKs. However, the greater efficacy against STAT2/3 suggests an additional, specific mechanism, such as preferential binding to their SH2 domains. This selectivity is a significant therapeutic advantage over broad JAK inhibitors, as it may preserve critical STAT1-mediated antimicrobial defenses while effectively dampening the more detrimental STAT2/3-driven inflammation. Collectively, LF-C3GNPs broadly suppress STAT signaling but demonstrate superior potency against the STAT2 and STAT3 pathways.

### 3.5 Effects of LF-C3GNPs on H_2_O_2_-induced ROS imbalance in HC11 cells

**Fig. 4:**
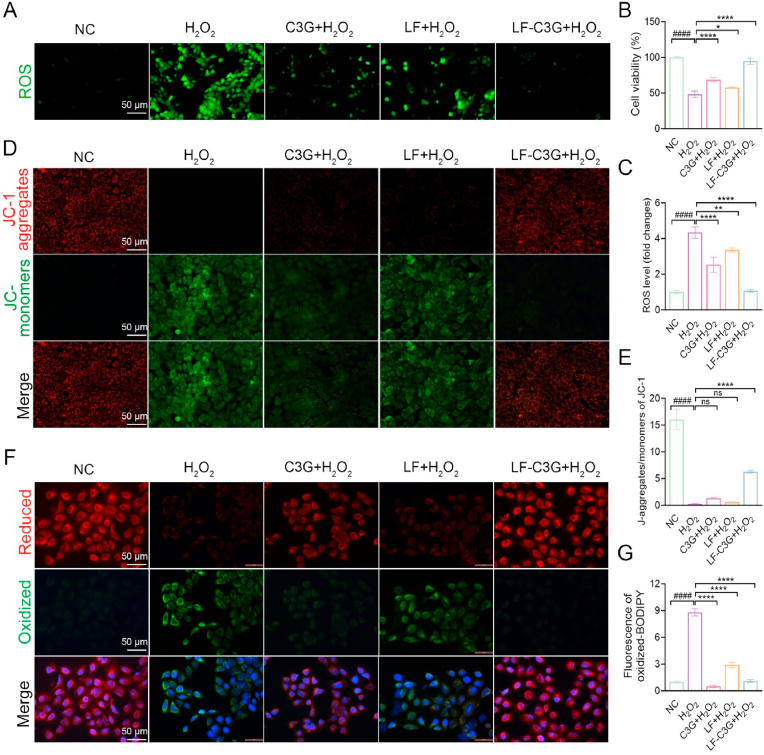
Effects of LF-C3GNPs on H_2_O_2_-induced ROS imbalance in HC11 cells. **(A)** ROS fluorescence images and **(C)** ROS quantification of HC11 cells treated with LF, C3G, and LF-C3GNPs, followed by H_2_O_2_ exposure, respectively (n = 3). **(B)** CCK-8 assay for cell viability of LF, C3G, and LF-C3GNPs. **(D, E)** JC-1 staining to assess mitochondrial membrane potential (ΔΨm) **(D)** and corresponding fluorescence intensity ratios (red/green) across treatment groups **(E)** (Scale bar: 50 μm). **(F, G)** Lipid peroxidation visualized via C11-BODIPY^581/589^-staining **(F)** after different treatments and quantitative analysis of oxidative membrane damage **(G)** (Scale bar: 50 μm). Mean ± SD; **^#^***p<0.05* compared to the untreated control group; **∗***p<0.05*, **∗∗***p<0.01*, **∗∗∗***p<0.001*, and **∗∗∗∗***p<0.0001* relative to the H_2_O_2_ -treated groups. (For interpretation of the references to colour in this figure legend, the reader is referred to the Web version of this article.)

Oxidative stress significantly contributes to the development of mastitis, stemming from an imbalance between mammary antioxidant defenses and excessive ROS production driven by increased metabolic activity, which negatively impairs lactation in women [7]. We therefore intended to explore the effects of LF-C3GNPs on oxidative stress mediated by ROS imbalance in HC11 cells. Hydrogen peroxide (H_2_O_2_), one of the most common endogenous ROS types widely used to induce oxidative stress in biological systems, was applied at 400 mM to simulate oxidative damage. HC11 cells were pretreated with graded concentrations of LF, free C3G, and LF-C3GNPs for 2 h, followed by a 24 h exposure to H_2_O_2_. CCK-8 assays indicated a significant decline in viability in H_2_O_2_-treated cells, while partial restoration of viability was observed in cells treated with LF or free C3G. Notably, pretreatment with LF-C3GNPs effectively restored cell viability to levels comparable to untreated controls (Fig. 4B).

Excessive ROS, predominantly generated in mitochondria [64], can trigger oxidative stress and mitochondrial dysfunction. To evaluate intracellular ROS levels in HC11 cells following H_2_O_2_ exposure, we employed DCFH-DA fluorescence staining. H_2_O_2_-treated cells increased ROS levels, which were significantly attenuated by LF-C3GNPs pretreatment relative to the levels observed in LF or free C3G. (Fig. 4A–C). The ROS-scavenging capacity of phenolic phytochemical compounds such as C3G stems from their phenolic hydroxyl groups, which donate hydrogen atoms to stabilize free radicals [65]. This suggests that LF-C3GNPs exhibit strong ROS-scavenging and cytoprotective effects against H_2_O_2_-induced oxidative imbalance. Given the central role of mitochondria in ROS generation [66,67], we further explored mitochondrial membrane potential (ΔΨm) using JC-1 staining. H_2_O_2_ exposure significantly decreased ΔΨm, indicating mitochondrial depolarization and dysfunction. However, LF-C3GNPs effectively restored ΔΨm closer to normal control than LF or free C3G treatments (Fig. 4D and E). These findings confirm that LF-C3GNPs mitigate ROS imbalance-induced oxidative stress by improving mitochondrial integrity and functionality in HC11 cells.

Lipid peroxidation, an irreversible biochemical process signaling necrotic cell death, often arises as a terminal outcome of oxidative damage [68]. To assess the effect of LF-C3GNPs on this process, the lipophilic fluorescent probe BODIPY C-11^581/589^ was employed as a ratiometric probe. Results revealed heightened oxidative damage in H_2_O_2_-exposed cells, whereas LF-C3GNPs significantly reduced H_2_O_2_-induced lipid peroxidation than LF or free C3G (Fig. 4F and G). Consistently, LF-C3GNPs substantially enhanced the cellular defense system by restoring ΔΨm activity and reducing membrane lipid peroxidation, thereby mitigating ROS imbalance and retaining redox homeostasis.

### **3.6** Effect of LF-C3GNPs on H_2_O_2_-induced oxidative stress and inflammation in HC11 cells via *Sesn2/Nrf2* signaling activation

**Fig. 5:**
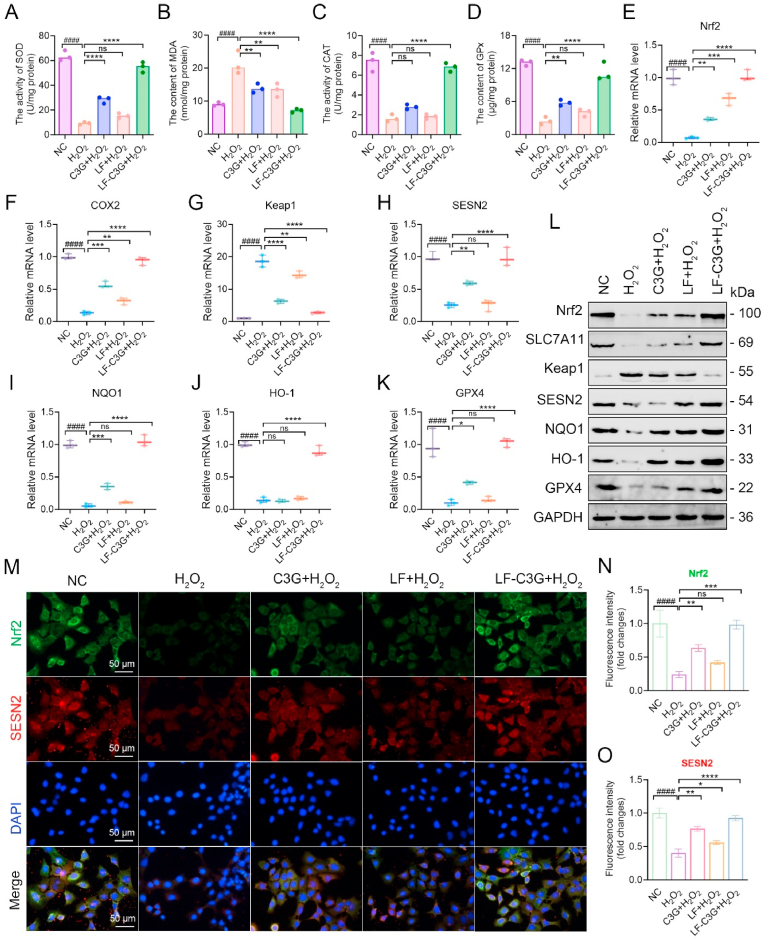
Effect of LF-C3GNPs on H_2_O_2_-induced oxidative stress and inflammation in HC11 cells via *Sesn2/Nrf2* signaling activation. **(A**–**D)** Antioxidant enzyme activity of SOD, CAT, GPx and lipid peroxidation marker (MDA) levels in HC11 cells (n = 3). **(E**–**L)** RT-qPCR analysis **(E**–**K)** and Western blotting **(L)** of key redox regulators biomarkers (Nrf2, SLC7A11, Keap1, Sesn2, NQO1, HO-1, and GPX4) under different treatments (n = 3). **(M**–**O)** Immunofluorescence staining **(M)** and quantification **(N, O)** of Nrf2 and Sesn2 in HC11 (n = 3). Scale bar: 50 μm. Mean ± SD; **^#^***p<0.05* compared to the untreated control group; **∗***p<0.05*, **∗∗***p<0.01*, **∗∗∗***p<0.001*, and **∗∗∗∗***p<0.0001* relative to the H_2_O_2_ -treated groups.

Research has shown that inflammation and oxidative stress are intricately connected and concurrently reinforcing mechanisms in the pathogenesis of mastitis [42]. Excessive generation of ROS by inflammatory cells plays a vital role in driving oxidative stress and activating signaling pathways that upregulate pro-inflammatory gene expression [69,70]. While ROS production is essential for pathogen clearance during inflammation, its persistent overproduction exacerbates oxidative damage and triggers various transcription factors that alter the expression of genes evolved in inflammation [70]. To address this, we investigated the effects of LF-C3GNPs on ROS-mediated oxidative stress and inflammation in HC11 cells, with a focus on the *Sesn2/Nrf2* signaling. Notably, Oxidative stress is typically evaluated by measuring antioxidant enzyme activity (SOD, CAT, GPx) to assess cellular defense mechanisms and malondialdehyde (MDA) concentration to quantify lipid peroxidation. H_2_O_2_ exposure substantially increased malondialdehyde (MDA) expression levels while suppressing expression of antioxidant enzymes (SOD, CAT, GPx). Disruption in the balance between ROS generation and the cellular antioxidant defense mechanisms initially triggers HC11 cell damage, which may progress to epithelial dysfunction of the mammary gland. LF-C3GNPs treatment reversed these trends relative to LF or free C3G-treated groups, counteracting enzymatic depletion and lipid damage (Fig. 5A–D), suggesting a substantially enhanced cellular antioxidant defense system.

Notably, H_2_O_2_ exposure induced significant oxidative stress, evidenced by the downregulation of Nrf2, Sesn2, GPX4, HO-1, SLC7A11, and NQO1 at the mRNA and protein levels, along with an increase in Keap1 expression. Treatment with LF-C3GNPs significantly reversed these H_2_O_2_-induced effects, demonstrating a more potent efficacy than LF or free C3G (Fig. 5E–L and Fig. S3). This data indicates that LF-C3GNP treatment promotes Nrf2 activation. As a primary transcriptional regulator of antioxidant response elements (ARE), activated Nrf2 increases the expression of downstream genes, including HO-1, NQO1, GPX4, and SLC7A11. This aligns with established research on the protective role of the Nrf2/HO-1 signaling [71]. The subsequent upregulation of the antioxidant enzymes NQO1's suppression of cytokines [72], SLC7A11's role in cysteine transport, and GPX4's neutralization of lipid peroxides collectively enhances the cellular capacity to clear ROS and inhibit the transcription of pro-inflammatory mediators [73]. These results demonstrate that LF-C3GNPs enhance cellular antioxidant defenses primarily through Nrf2 activation, thereby mitigating oxidative stress-induced inflammation.

Sesn2 and Nrf2 are critically involved in oxidative and inflammatory pathologies, as their upregulation suppresses pro-inflammatory cytokines and provides therapeutic benefits [74]. We found that LF-C3GNPs potently activate *Sesn2/Nrf2* signaling to oppose ROS-driven stress and inflammation. While H_2_O_2_ reduced Sesn2 and Nrf2 fluorescence signals, LF-C3GNPs enhanced their expression more effectively than other treatments (Fig. 5M–O). This suggests that Sesn2 increases under stress conditions and helps control ROS overproduction, complementing Nrf2's established role in redox balance [33]. Therefore, LF-C3GNPs offer a dual mechanism against oxidative stress. They appear to counteract the ROS-induced disruption of the Keap1-Nrf2 system by fostering a reinforcing activation cycle between Sesn2 and Nrf2 [73], which stimulates antioxidant genes and ROS scavenging. This synergistic regulation mitigates oxidative stress-driven inflammation, as demonstrated in a mastitis model, highlighting the therapeutic promise of LF-C3GNPs for similar conditions.

### 3.7 Effects of LF-C3GNPs on *S. aureus*-induced inflammation in mice

**Fig. 6:**
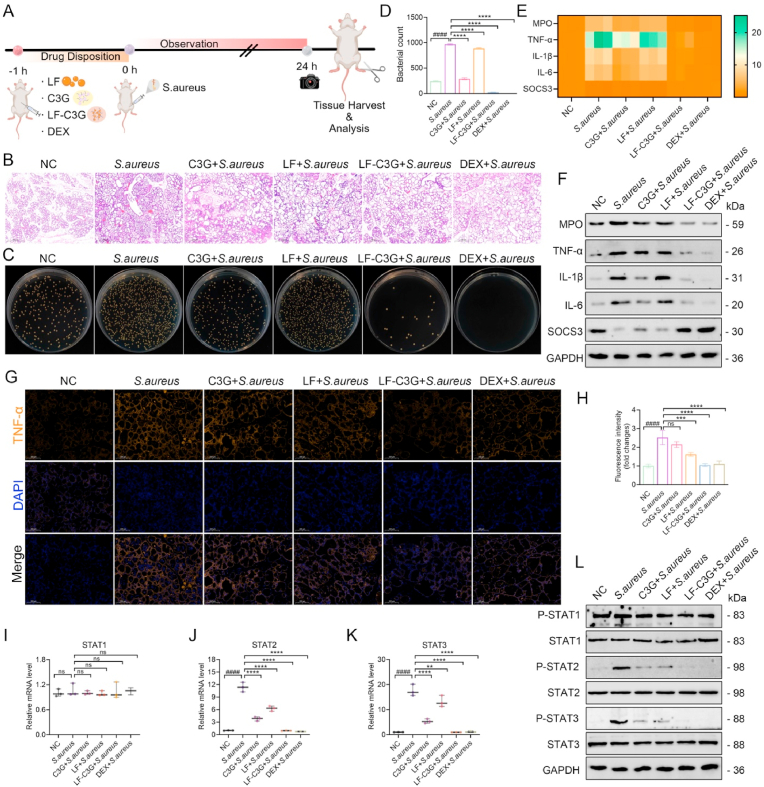
Effects of LF-C3GNPs on *S. aureus*-induced inflammation in mice. **(A)** Schematic diagram of mastitis modeling and LF-C3GNPs pre-administration in *S. aureus*-induced mastitis in mice. **(B)** H&E assay changes of *S. aureus*-infected mammary tissues in different groups. Scale bar: 500 μm. **(C)** Bacterial colony-forming unit (CFU) on agar plates and **(D)** quantification of CFU *after different treatment* groups. **(E)** Heatmap quantification and **(F)** proteins expression levels of MPO, TNF-α, IL-1β, IL-6, and SOCS3 in infected mice CFU *after different treatment* groups. **(G)** Immunofluorescence staining and **(H)** quantification of TNF-α. Scale bar: 200 μm. **(I**–**K)** mRNA and **(L)** Protein expression levels of phosphorylated STATs (p-STAT1, p-STAT2, and p-STAT3) and STAT1, STAT2, and STAT3 in infected mice. Mean ± SD; **^#^***p<0.05* compared to the untreated control group; **∗***p<0.05*, **∗∗***p<0.01*, **∗∗∗***p<0.001*, and **∗∗∗∗***p<0.0001* relative to the *S. aureus*-treated groups.

Inspired by the strong anti-inflammatory effect of LF-C3GNPs *in vitro*, we further explored their therapeutic potential in a pregnant mouse model of *S. aureus*-induced inflammation designed to mimic human mastitis. The use of a pregnant mouse model to mimic human mastitis is clinically significant (Fig. 6A), as mastitis frequently occurs during lactation and poses risks to both maternal and infant health [42]. The effective *in vivo* dose (50 mg/kg) of C3G, LF, and LF-C3GNPs was *administered* intravenously to the mice, which translates to a human equivalent dose (HED) of approximately 4 mg/kg. This HED is considered clinically feasible, supported by the established safety profile of C3G and the GRAS (Generally Recognized As Safe) status of lactoferrin (LF). H&E staining revealed neutrophil infiltration in interstitial spaces and alveolar lumina of infected mammary glands (Fig. 6B). *S. aureus* infection induced alveolar wall degradation, epithelial cell necrosis, vascular congestion, and dense inflammatory cell clusters in alveolar spaces, validating hallmark features of clinical mastitis. Notably, administration of LF-C3GNPs substantially mitigated hyperemia and the influx of inflammatory cells relative to treatments with LF or free C3G. The groups treated with DEX, a potent glucocorticoid used as a positive control, exhibited results similar to those of LF-C3GNPs. This is noteworthy, as long-term DEX use is associated with immunosuppression and metabolic side effects, whereas LF-C3GNPs may offer a safer alternative application. These results showed that LF-C3GNPs enabled delivery to achieve DEX-comparable anti-inflammatory efficacy without compromising safety.

Mastitis is a common cause of *S. aureus*-induced mammary gland infections, which can compromise tissue function. The resistance of *S. aureus* to traditional antibiotics is a significant challenge in pathogen-driven inflammatory diseases, such as mastitis, highlighting the need for innovative therapies [5]. Therefore, exploring the *in vivo* effectiveness of LF-C3GNPs is essential for strengthening the host's defenses against mastitis. In *S. aureus*-infected mice, bacterial colony counts from mammary tissues revealed substantial bacterial loads. However, LF-C3GNPs significantly reduced bacterial loads and mitigated tissue pathology relative to LF or free C3G treatments (Fig. 6C and D). LF-C3GNPs demonstrated antibacterial effects slightly similar to those of the DEX group, outperforming most traditional antibiotics *in vivo*. They leverage LF's ability to deprive pathogens of iron, a nutrient critical for biofilm formation, while C3G directly disrupts bacterial membranes.

This mechanism works synergistically to enhance immunomodulatory activity, thereby strengthening the body's innate tissue defenses against bacterial pathogens. This finding is supported by further research demonstrating that a complex formulation containing C3G yielded potent antibacterial effects [75,76], unlike curcumin- or EGCG-based nanoformulations [12]. Consistent with H&E staining results, LF-C3GNPs demonstrate targeted accumulation in inflamed mammary tissue, enhancing localized efficacy while reducing systemic exposure. This dual functionality, combining antibacterial and anti-inflammatory effects, addresses the critical challenge of infections like mastitis, where *S. aureus*-induced inflammation exacerbates tissue damage. By integrating pathogen-targeting and host-protective mechanisms, LF-C3GNPs represent a superior therapeutic strategy for managing complex infections compared to traditional nanoformulations.

Myeloperoxidase (MPO) activity, a critical biomarker of neutrophil infiltration [42,54], was substantially reduced in *S. aureus*-infected mammary tissues following LF-C3GNPs pretreatment. LF-C3GNPs reduced neutrophil influx more effectively than LF or free C3G (Fig. 6E–F, Fig. S4A), demonstrating a superior ability to combat inflammation caused by neutrophil recruitment. Furthermore, the mammary tissues of infected mice exhibited upregulated mRNA and protein levels of the pro-inflammatory cytokines TNF-α, IL-1β, and IL-6, concurrent with a reduction in SOCS3 expression compared to control. However, LF-C3GNPs administration substantially suppressed these inflammatory mediators relative to LF, free C3G, or DEX treatments (Fig. 6E, F, and Fig. S4B–E). Given TNF-α′s central role in mediating inflammatory responses and activating downstream cytokines, immunofluorescence staining of mammary tissues revealed heightened TNF-α fluorescence intensity in infected mice, whereas LF-C3GNPs or DEX-treated groups showed significantly decreased TNF-α staining (Fig. 6G and H). These results highlight the dual capacity of LF-C3GNPs to reduce cytokine storms and neutrophil infiltration, offering a multifaceted therapeutic approach in *S. aureus*-induced mastitis.

Building on the *in vitro* results, we investigated whether LF-C3GNPs could also reduce STAT3, STAT2, and STAT1 activity, key regulators of pro-inflammatory cytokine and enzyme production, in *S. aureus*-infected mammary tissues. Results showed that infection with *S. aureus* substantially increased phosphorylated STAT3 (p-STAT3) and STAT2 (p-STAT2) expression levels. Administration of LF-C3GNPs or DEX significantly reversed this phosphorylation (Fig. 6I–L, Fig. S5), underscoring their role in modulating downstream JAK-STAT signaling. STAT1 and STAT3/STAT2 exhibit distinct structural conformations in their SH2 domains, essential for phosphorylation and dimerization [61]. LF-C3GNPs may interact with residues unique to STAT2/STAT3, blocking their activation while sparing STAT1. STAT1 activation is tightly regulated by SOCS1, whereas STAT3/STAT2 are modulated by SOCS3 [62]. LF-C3GNPs C3G's ability to upregulate SOCS3 (Fig. 6E, F, and Fig. S4E) may selectively dampen STAT3/STAT2 signaling. Accordingly, the activation of STAT3 and STAT2 signaling pathways drives the release of cytokines, mobilizing immune cells such as neutrophils, monocytes, and macrophages (M1) to infiltrate the mammary gland, a key pathological feature of mastitis. This signaling cross-talk between mammary epithelial cells and macrophages is crucial in mitigating *S. aureus* infection within the mammary tissue [5]. These results correlate strongly with reduced MPO activity (Fig. 6E, F, and Fig. S4E), substantiating the anti-inflammatory activity. Similarly, research has shown that inflammatory cytokines orchestrate JAK-STAT signaling in mammary tissue, culminating in the phosphorylation of STAT proteins that translocate to the nucleus, driving the transcription of pro-inflammatory genes and exacerbating the pathological mechanisms underlying mastitis [77]. Collectively, LF-C3GNPs significantly mitigated mammary gland inflammation by regulating JAK-STAT signaling pathways via STAT3 and STAT2 activation, offering a targeted therapeutic strategy against inflammatory diseases such as mastitis.

### **3.8** Effect of LF-C3GNPs on *S. aureus*-induced oxidative stress and inflammation in mice via *Sesn2/Nrf2* signaling activation

**Fig. 7:**
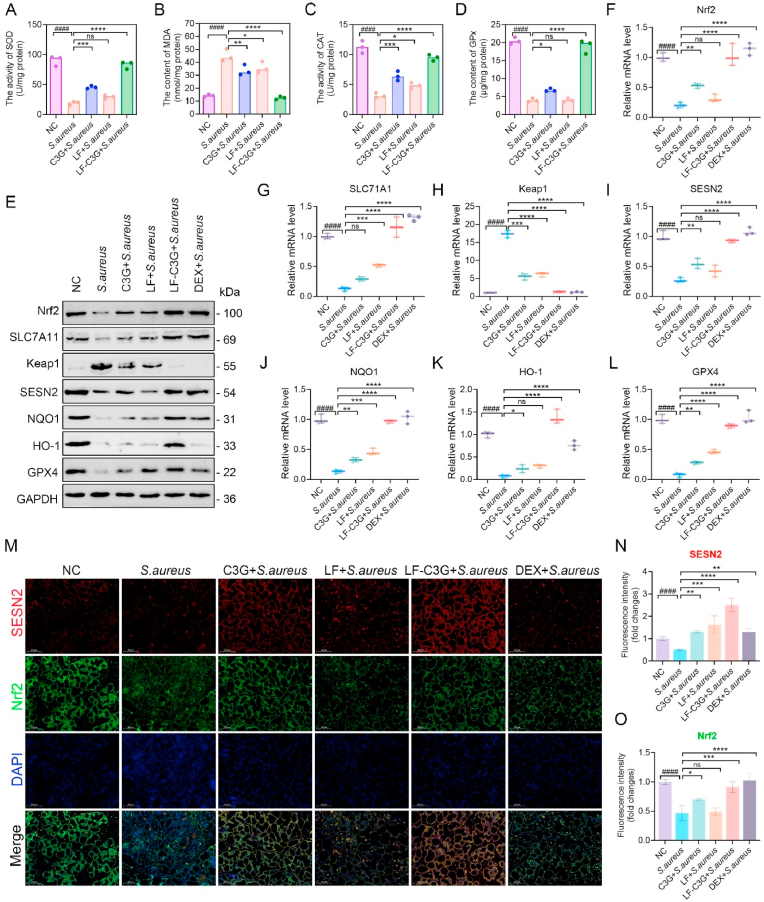
Effect of LF-C3GNPs on *S. aureus*-induced oxidative stress and inflammation in mice via *Sesn2/Nrf2* signaling activation**. (A**–**D)** Antioxidant enzyme activity of SOD, CAT, GPx and lipid peroxidation marker (MDA) levels in *S. aureus*-infected mammary tissue (n = 3). **(E**–**L)** Western blotting **(E)** and RT-qPCR analysis **(F**–**L)** of key redox regulators biomarkers (Nrf2, Sesn2, Keap1, SLC7A11, NQO1, HO-1, and GPX4) under different treatments (n = 3). **(M**–**O)** Immunofluorescence staining **(M)** and quantification **(N, O)** of Nrf2 and Sesn2 in *S. aureus*-infected mammary tissue (n = 3). Scale bar: 50 μm. Mean ± SD; **^#^***p<0.05* compared to the untreated control group; **∗***p<0.05*, **∗∗***p<0.01*, **∗∗∗***p<0.001*, and **∗∗∗∗***p<0.0001* relative to the *S. aureus*-treated groups.

Emerging research underscores the critical role of antioxidant mediators in mitigating oxidative stress-driven inflammation processes, revealing their essential function in regulating immune responses [48]. Anthocyanin C3G, widely recognized for its potent antioxidant capabilities, demonstrated strong activity *in vitro* when formulated as LF-C3GNPs (Fig. 5A–D). To assess the therapeutic potential of LF-C3GNPs *in vivo*, we investigated the levels of key oxidative biomarkers, GPx, MDA, SOD, and CAT, in a murine model of *S. aureus*-induced mastitis. As expected, SOD, GPx, and CAT activities were significantly decreased in infected mice, while MDA levels surged compared to control mice (Fig. 7A–D), reflecting severe oxidative imbalance. Notably, LF-C3GNPs administration enhanced SOD, GPx, and CAT activity, while lowering MDA expression to levels close to those of normal control. Treatments with LF, free C3G, and DEX showed moderate trends. This suggests LF-C3GNPs' capacity to enhance enzymatic antioxidant activity that counteract *S. aureus*-induced oxidative damage in mammary tissues, likely through C3G's ROS-scavenging properties and LF's role in maintaining redox balance. Significantly, GPx, which contains sulfhydryl (-SH) groups linked to amino acids like glutamic acid, cysteine, and glycine, enhances antioxidant enzyme activity against oxidative stress. MDA, a byproduct generated during the terminal stages of lipid peroxidation, is a key biomarker for assessing oxidative damage severity. SOD neutralizes free radicals and ROS generated under stress, while CAT acts as a primary defense against superoxides, preventing cellular damage [75]. Together, these findings suggest that LF-C3GNPs effectively mitigate oxidative stress via strong antioxidant capabilities, alleviating mastitis pathology, which offers LF-C3GNPs as an innovative therapeutic approach for oxidative-inflammatory disorders.

To further explore the potential of LF-C3GNPs in mitigating oxidative stress and inflammation via the Sesn2/Nrf2 signaling pathway in a mouse model of *S. aureus*-induced mastitis, we conducted Western blotting and RT-qPCR analyses of key redox regulators, including Nrf2, Sesn2, Keap1, SLC7A11, NQO1, HO-1, and GPX4. Result revealed that *S. aureus* infection substantially suppressed mRNA and protein levels of these antioxidant biomarkers while increasing Keap1 expression (Fig. 7E–L, and Fig. S6). Conversely, LF-C3GNPs reversed these effects relative to LF, free C3G, and DEX treatments. Specifically, LF-C3GNPs upregulated Nrf2, which activated the Nrf2/HO-1 signaling, stimulating downstream antioxidant genes (NQO1, SLC7A11, GPX4) critical for countering oxidative stress-driven inflammation. Additionally, LF-C3GNPs simultaneously suppressed Keap1 and enhanced Sesn2 expression, promoting the disruption of the Keap1-Nrf2 complex. This facilitated activation of the Sesn2/Nrf2 signaling cascade, alleviating oxidative damage. Together, these findings demonstrate that LF-C3GNPs exert potent antioxidant effects by synergically modulating the Nrf2-ARE and *Sesn2/Nrf2* pathways, effectively reducing *S. aureus*-induced oxidative stress and inflammatory damage in mammary tissues.

Moreover, immunofluorescence double-staining of Sesn2 and Nrf2 in mammary tissues revealed decreased expression and reduced nuclear presence of both proteins in *S. aureus*-infected groups. Conversely, LF-C3GNPs treatment significantly enhanced cytoplasmic Nrf2 levels and increased Sesn2 signal intensity (Fig. 7M). Quantitative analysis further confirmed a pronounced rise in fluorescence intensity in the LF-C3GNPs-treated group relative to those observed in LF, free C3G, or DEX-treated groups (Fig. 7N and O). These findings confirm that LF-C3GNPs enhanced *Sesn2/Nrf2* signaling activation, facilitating Nrf2 translocation into the nucleus and stimulating transcriptional upregulation of downstream antioxidant genes. The synchronized upregulation of Sesn2 and Nrf2 underscores the mechanistic role of LF-C3GNPs in restoring redox homeostasis, effectively countering oxidative stress and inflammation in mastitis.

### **3.9** Biosafety of LF-C3GNPs *in vitro* and *in vivo* evaluation

**Fig. 8:**
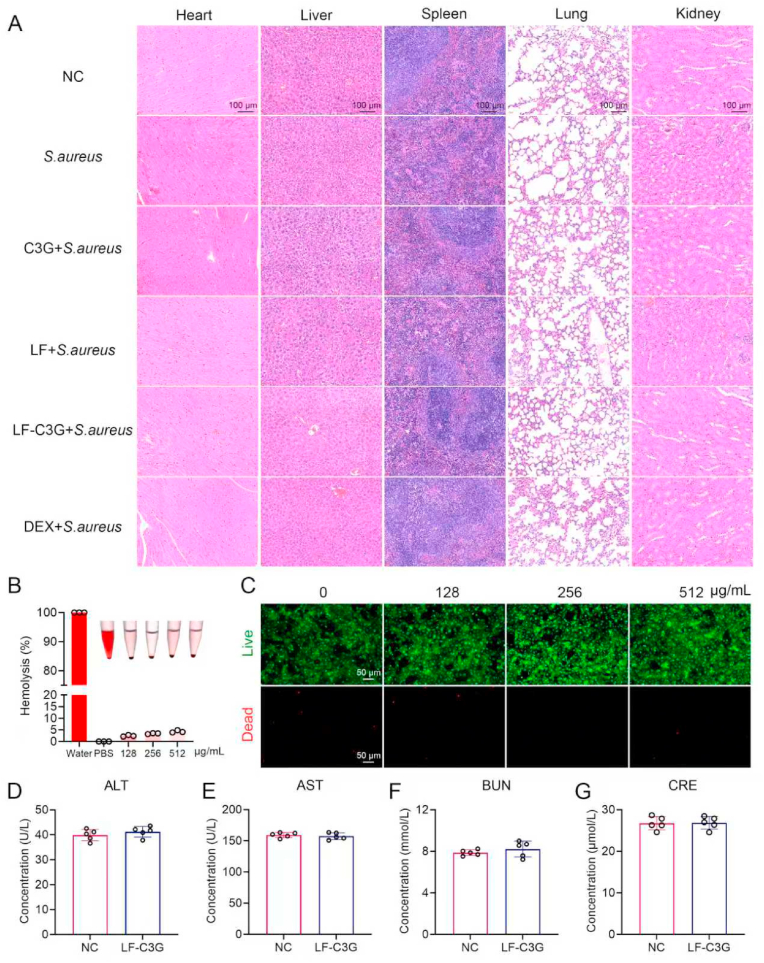
Biosafety of LF-C3GNPs *in vitro* and *in vivo* evaluation. **(A**) H&E-stained sections of the major organs (Heart, liver, spleen, lung, and kidneys) of mice with different treatments. Scale bar: 100 μm. **(B)** The hemolysis test result of LF-C3GNPs across tested concentrations (n = 3). **(C)** Fluorescence images of calcein AM and PI-stained HC11 cells exposed to LF-C3GNPs at different concentrations. Scale bar = 50 μm. **(D**–**G)** Blood biochemistry results of ALT, AST, BUN, and CRE in control and LF-C3GNPs-treated mice (n = 5).

Ensuring biosafety is paramount for transitioning nanomaterials into clinical biomedical applications [78]. Building on *in vitro* cytotoxicity data showing over 90 % cell viability for LF, C3G, and LF-C3GNPs (Fig. 3A–C), we assessed the *in vivo* systemic toxicity in a mouse model of *S. aureus*-induced mastitis. Mice received LF, C3G, or LF-C3GNPs (50 mg/kg) for 24 h (Fig. 5A). H&E staining of major organs showed no pathology. Cardiac myofibrils (heart), hepatic sinusoids and hepatocytes (liver), red/white pulp (spleen), alveolar architecture (lungs), and glomeruli/tubules (kidneys) remained intact without necrosis, congestion, or leukocyte infiltration in LF-C3GNPs-treated groups (Fig. 8A). The lack of histopathological alterations in vital organs confirms LF-C3GNPs’ biocompatibility *in vivo*, with no evidence of systemic toxicity or organ impairment.

The LF-C3GNPs administration in the *S. aureus*-induced mastitis mouse model highlights two critical advances, including excellent biocompatibility and enhanced therapeutic efficacy at a 50 mg/kg dose over 24 h. LF-C3GNPs significantly mitigated off-target effects relative to the moderate attenuation shown in LF, free C3G, or DEX-treated groups. LF-C3GNPs' biocompatibility likely stems from lactoferrin's natural origin and its role in reducing oxidative stress, combined with C3GNPs-mediated controlled release, which minimizes burst effects and tissue injury. The lack of organ impairment supports LF-C3GNPs as a viable candidate for *in vivo* applications, addressing a common hurdle in nanomedicine where carrier materials often induce unintended immune or toxic responses. Furthermore, the significant short-term therapeutic effects demonstrate enhanced bioavailability and tissue penetration. This suggests that the LF-C3GNPs likely protect C3G from rapid clearance or degradation, prolonging its circulation time and enabling sustained delivery to infected tissues. Lactoferrin's intrinsic antimicrobial and immunomodulatory properties may amplify C3G's anti-biofilm and antioxidant effects, creating a dual-action therapeutic. The dose-dependent efficacy of LF-C3GNPs relative to LF, free C3G, or DEX implies that nanoencapsulation optimizes pharmacokinetics, potentially reducing the effective dose required for clinical outcomes. This is critical for minimizing long-term toxicity risks.

Moreover, hemolysis assays, a standard method for assessing hemocompatibility of biomaterials *in vitro*, demonstrated that LF-C3GNPs induced hemolysis rates below 5 % across various concentrations (Fig. 8B), consistent with safety thresholds established for nanomaterials [79]. Even at a high dose of 512 μg/mL, LF-C3GNPs exhibited negligible erythrocyte damage compared to controls, underscoring their excellent hemocompatibility. This suggests LF-C3GNPs as promising candidates for parenteral drug delivery. Similarly, Calcein-AM/PI viability assays further confirmed the superior cytocompatibility of LF-C3GNPs. Fluorescence imaging revealed predominantly green signals (live cells) and minimal red fluorescence (dead cells) in all LF-C3GNPs-treated groups, even at a high concentration of 512 μg/mL (Fig. 8C). This evidence confirms that LF-C3GNPs do not trigger significant apoptosis or necrosis, reinforcing the results from CCK-8 assays and H&E staining, showing that the nanoencapsulation is non-toxic and safe for biomedical applications.

Additionally, we expanded the investigation to include biochemical indicators of liver and kidney function, such as ALT, AST, BUN, and CRE in LF-C3G groups. The results showed that LF-C3GNPs lowered levels of these markers, indicating no significant hepatotoxicity or nephrotoxicity and supporting the biocompatibility of LF-C3GNPs (Fig. 8D–G). This further emphasizes its systemic protective effects against liver and kidney injury. The stability of these biochemical indicators is crucial for therapeutic agents, especially in inflammatory diseases, such as mastitis, where systemic inflammation often affects multiple organs. The synergistic stability and bioavailability of LF-C3GNPs ensure sustained release, unlike existing nanoformulations, such as EGCG nanoparticles, which tend to degrade quickly in the oxidative environment of the mammary gland. Overall, the lack of systemic toxicity to major organs and hemolytic activity highlights the potential of LF-C3GNPs as a safe option for systemic use. These findings support preclinical safety standards focused on minimizing off-target organ damage. They could also promote further research into LF-C3G for therapeutic purposes, given its reassuring safety profile for vital organs like the liver and kidneys.

### **3.10** Evaluation of long-term biosafety of LF-C3GNPs *in vivo*

A comprehensive assessment of the long-term biocompatibility of LF-C3GNPs was conducted using a mouse model, integrating both histopathological and serum biochemical analysis. Following the administration of LF-C3GNPs, major organs—including the heart, liver, spleen, lungs, and kidneys—were harvested at 7-day and 14-day intervals for evaluation.

Histological analysis, conducted via H&E staining, revealed no signs of toxicity. All analyzed tissues retained their normal structural integrity, with distinct cellular borders and preserved nuclei at every time point (Fig. S7A). Notably, the organs exhibited no pathological findings such as lesions, necrosis, apoptotic activity, infiltration by inflammatory cells, or fibrosis. This absence of damage indicates that LF-C3GNPs did not induce acute or subacute tissue injury. The vital organ's well-preserved architecture after a 14-day treatment period strongly underscores the exceptional histocompatibility of the nanocomposite. This suggests that LF-C3GNPs are efficiently managed by the reticuloendothelial system without accumulating to toxic levels or triggering a harmful immune reaction that could result in chronic inflammation or impaired organ function.

To further quantitatively assess systemic toxicity, key serum biomarkers for liver and kidney health were analyzed. The concentrations of ALT and AST, established indicators of liver damage, remained within the normal physiological range after 7 and 14 days of treatment, showing no notable increase compared to the control (Fig. S7B and C). Likewise, the renal function markers blood BUN and CRE were unaltered by the prolonged administration of LF-C3GNPs (Fig. S7D and E). The stability of these critical clinical chemistry parameters confirms a lack of hepatotoxic or nephrotoxic effects. These biochemical results provide robust, quantitative support for the histological observations. The stable levels of ALT and AST confirm that LF-C3GNPs do not induce hepatocellular damage or compromise liver integrity. The liver, as a primary organ for metabolism and clearance, is often a site of nanoparticle accumulation; the lack of toxicity is particularly significant. Furthermore, the consistent BUN and CRE levels indicate a normal glomerular filtration rate and kidney function, mitigating concerns about potential nephrotoxicity, a frequent challenge for systemically administered agents.

The compelling biosafety data from this study, demonstrated by the concordance between unaltered tissue histology and stable biochemical markers, is a key finding. This implies that the synthesis and composition of LF-C3GNPs enhance their biocompatibility and reduce the risk of off-target toxicities common with synthetic nanocarriers. The demonstrated safety over two weeks supports the feasibility of repeated dosing regimens that may be required for treating chronic inflammatory conditions or persistent infections. Collectively, this thorough long-term biosafety evaluation confirms that LF-C3GNPs exhibit excellent *in vivo* tolerability with no evidence of hepatic, renal, or systemic toxicity. This strong safety profile is integral to their therapeutic potential and supports further promise for advancement into future preclinical and clinical trials. Subsequent research should focus on longer-term exposure studies and detailed investigations into the biodistribution and clearance mechanisms of LF-C3GNPs to further validate their safety.

## 4 Conclusion

This study evidenced that the imbalance in ROS induced by LTA and H_2_O_2_ triggers oxidative stress and inflammatory responses, disrupting cellular homeostasis and leading to substantial damage in HC11 cells and mammary tissues. Addressing the dual challenges of inflammation and oxidative stress in mastitis requires innovative therapeutic strategies. This study demonstrates that LF-C3GNPs effectively stabilize C3G, enhancing its bioavailability and therapeutic efficacy. By activating the *Sesn2/Nrf2* pathway, LF-C3GNPs restore redox balance and suppress inflammatory signaling in both cellular and animal models. The nanoparticles' capability to reduce bacterial load and tissue damage *in vivo*, along with their favorable biosafety attributes, makes them a potentially promising candidate for clinical translation. Notably, LF-C3GNPs demonstrated a superior therapeutic effect, integrating both anti-inflammatory and antioxidant activities, compared to LF, free C3G, or DEX, highlighting their enhanced performance over standard therapies. These findings advance nanomedicine strategies for mastitis and further highlight the potential of protein-phytochemical encapsulated NPs in addressing oxidative stress-driven inflammatory diseases. Future research should explore scalable synthesis and long-term safety to accelerate their transition into clinical practice. The detailed biodistribution and clearance pathways of LF-C3GNPs should further solidify their safety credentials.

## CRediT authorship contribution statement

**Fructueux Modeste Amona:** Writing – original draft, Validation, Investigation, Data curation. **Yipeng Pang:** Writing – review & editing, Validation, Data curation. **Xiaohan Chen:** Validation, Investigation, Data curation. **Zilu Liu:** Validation, Investigation, Data curation. **Chenyang Su:** Validation, Data curation. **Jiachen Yang:** Validation, Data curation. **Kejun Liu:** Validation, Data curation. **Qiyu Wu:** Validation, Data curation. **Bingbing Liu:** Validation, Data curation. **Xi Chen:** Writing – review & editing, Supervision, Funding acquisition, Conceptualization. **Chunlei Zhang:** Writing – review & editing, Supervision, Funding acquisition, Conceptualization.

## Funding

This research was funded by the 10.13039/501100001809National Natural Science Foundation of China (No. 32072712, 32000108), Basic Research Program of Jiangsu Province (BK20251929), 10.13039/501100004608Natural Science Foundation of Jiangsu Province (No. BK 20201022), the 10.13039/100014103Key Research and Development Plan (Modern Agriculture) Project of Xuzhou City (No. KC22076), and Natural Science Research of 10.13039/501100010023Jiangsu Higher Education Institutions of China (20KJB180006).

## Declaration of competing interest

The authors declare that they have no known competing financial interests or personal relationships that could have appeared to influence the work reported in this paper.

## Data Availability

Data will be made available on request.
