# Supplementary material for: Lactoferrin-cyanidin-3-glucoside nanoparticles alleviate inflammation and oxidative stress via Sesn2/Nrf2 activation in mastitis
Source: Mater Today Bio. 2025 Oct 31;35:102491. doi: 10.1016/j.mtbio.2025.102491 (PMC12637076; doi:10.1016/j.mtbio.2025.102491)
Supplement: Multimedia component 1 [file mmc1.docx]

**Supporting Information**


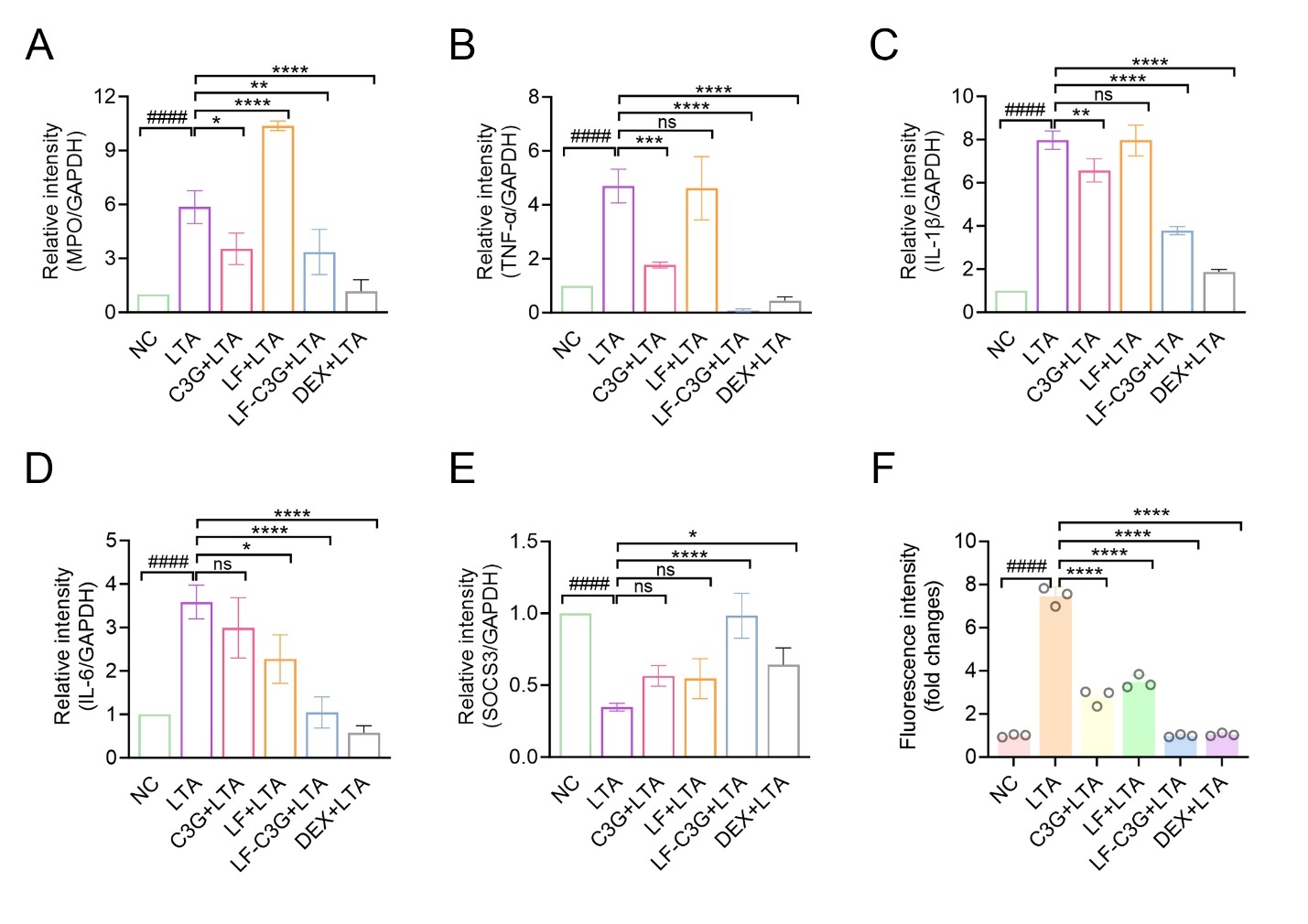


**Figure S1**. (**A-E**) Quantitative analysis of MPO, TNF-α, IL-1β, IL-6, and SOCS3 in HC11 cells. (**F**) Quantification of TNF-α immunofluorescence intensity in HC11 cells. Data are presented as mean ± standard deviation (SD) from three independent replicates. Statistical significance is designated as **^#^***p<0.05* compared to the untreated control group; ******p<0.05*, *******p<0.01*, and ********p<0.001* relative to the LTA-treated groups.


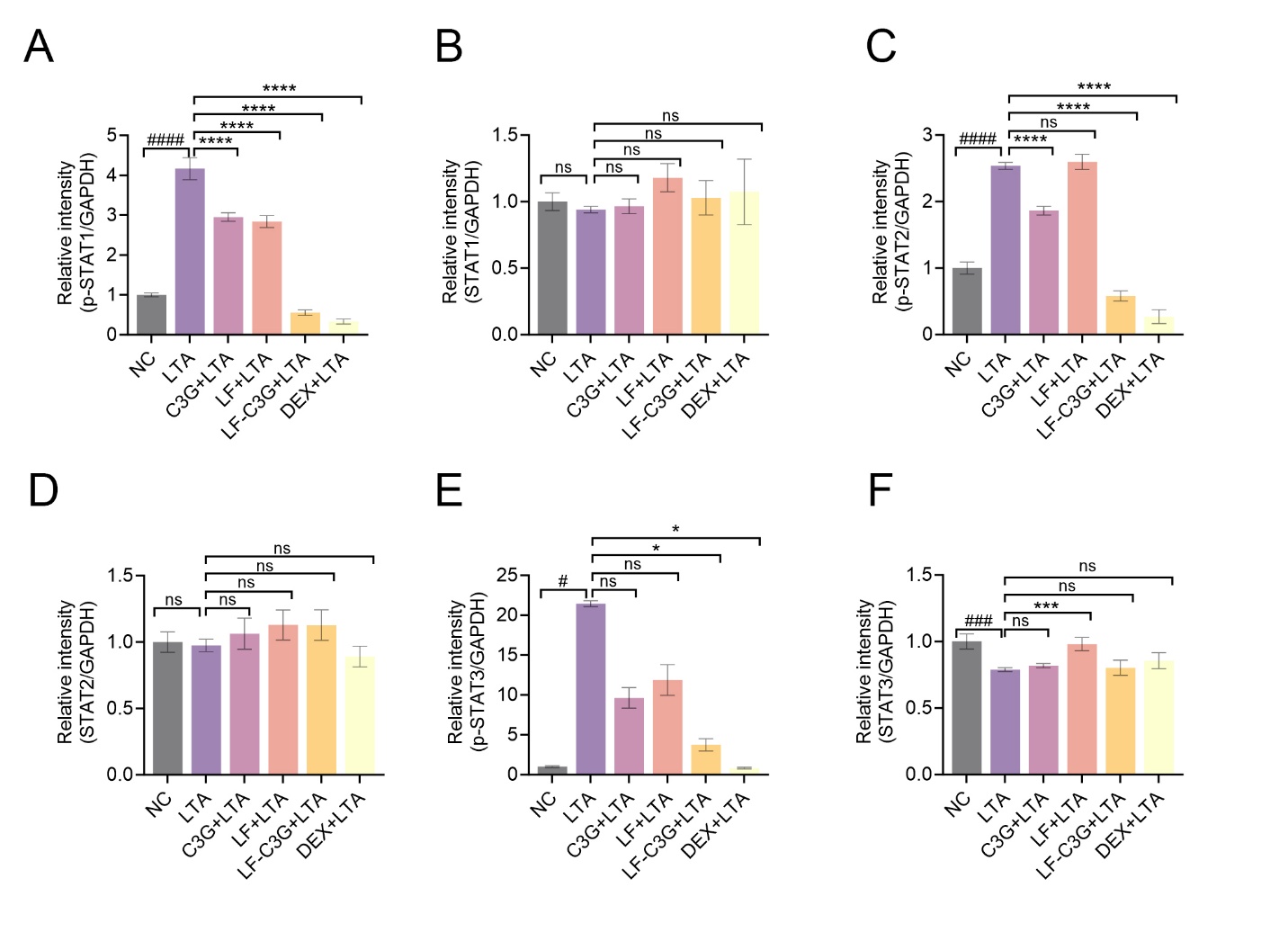


**Figure S2**. Quantitative analysis of phosphorylated STATs (p-STAT1, p-STAT2, and p-STAT3) and STAT1, STAT2, and STAT3 in HC11 cells after different treatments. Mean ± SD; **^#^***p<0.05* compared to the untreated control group; ******p<0.05*, *******p<0.01*, and ********p<0.001* relative to the **H_2_O_2_** -treated groups.


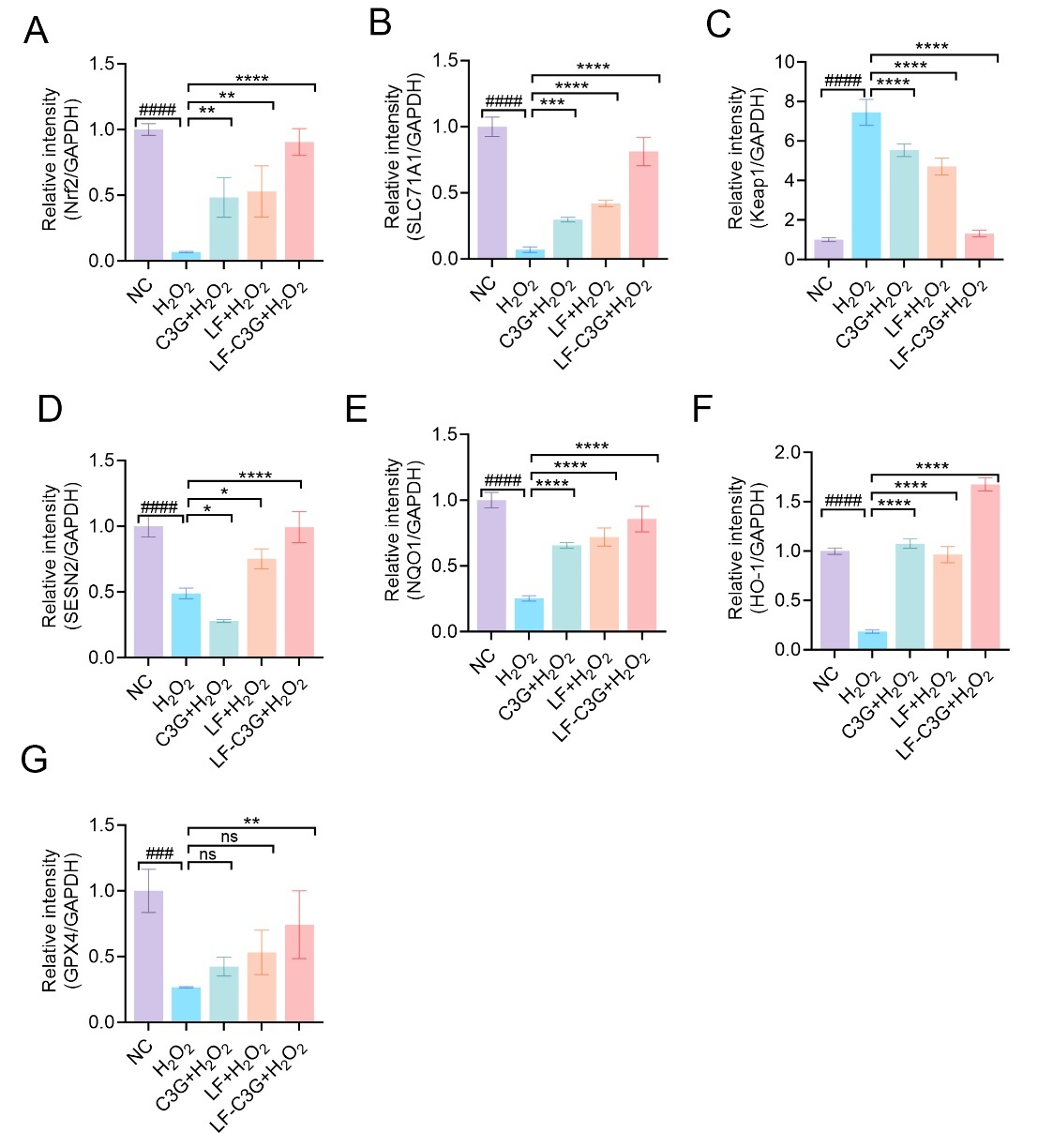


**Figure S3**. Quantitative analysis of Nrf2, Sesn2, Keap1, SLC7A11, NQO1, HO-1, and GPX4 under different treatments. Mean ± SD; **#***p<0.05* compared to the untreated control group; ******p<0.05*, *******p<0.01*, and ********p<0.001* relative to the **H_2_O_2_** -treated groups.


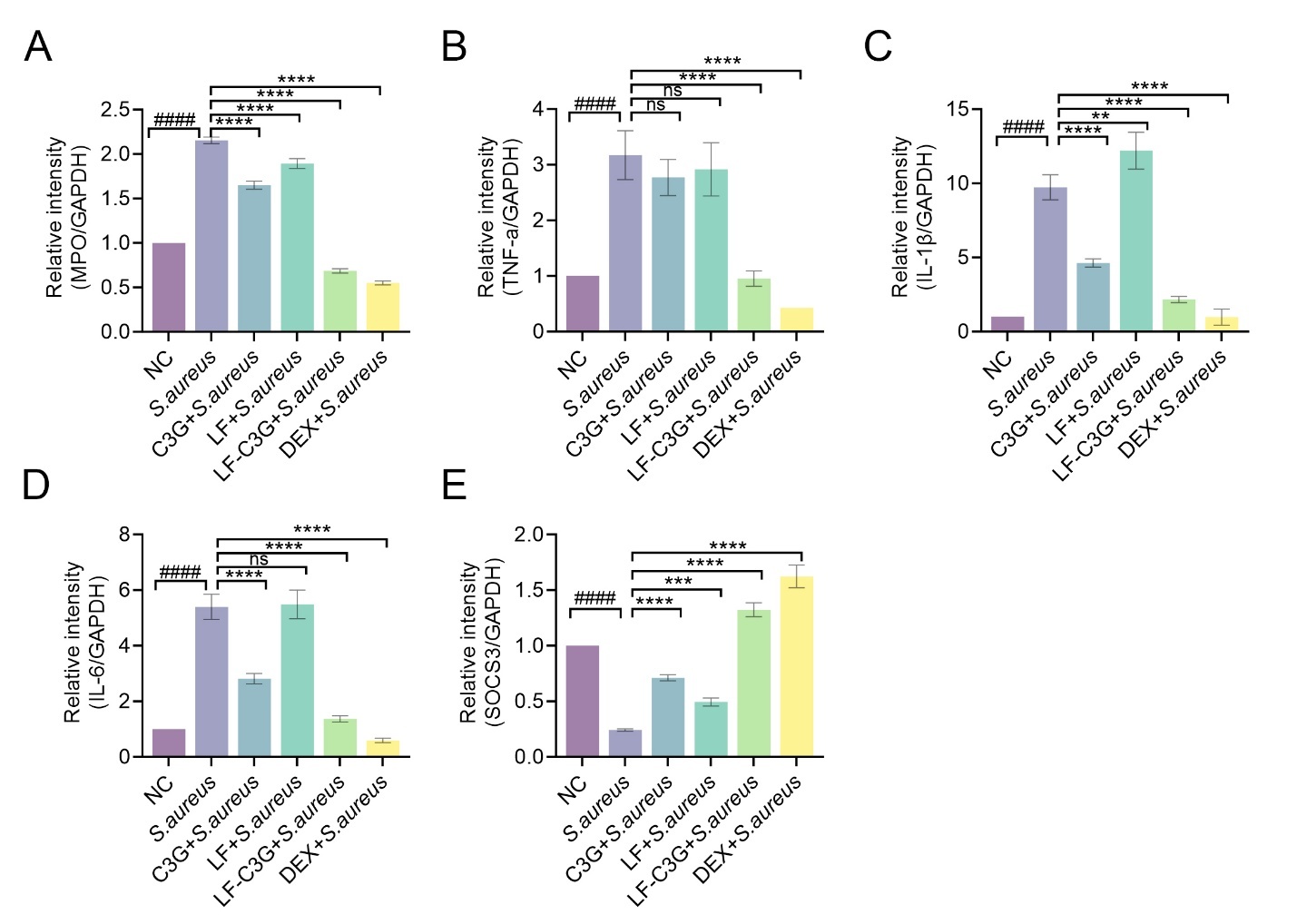


**Figure S4**. (A-C) Quantitative analysis of MPO, TNF-α, IL-1β, IL-6, and SOCS3 in ***S. aureus*-induced mice.** Mean ± SD; **#***p<0.05* compared to the untreated control group; ******p<0.05*, *******p<0.01*, and ********p<0.001* relative to the ***S. aureus***-treated groups.


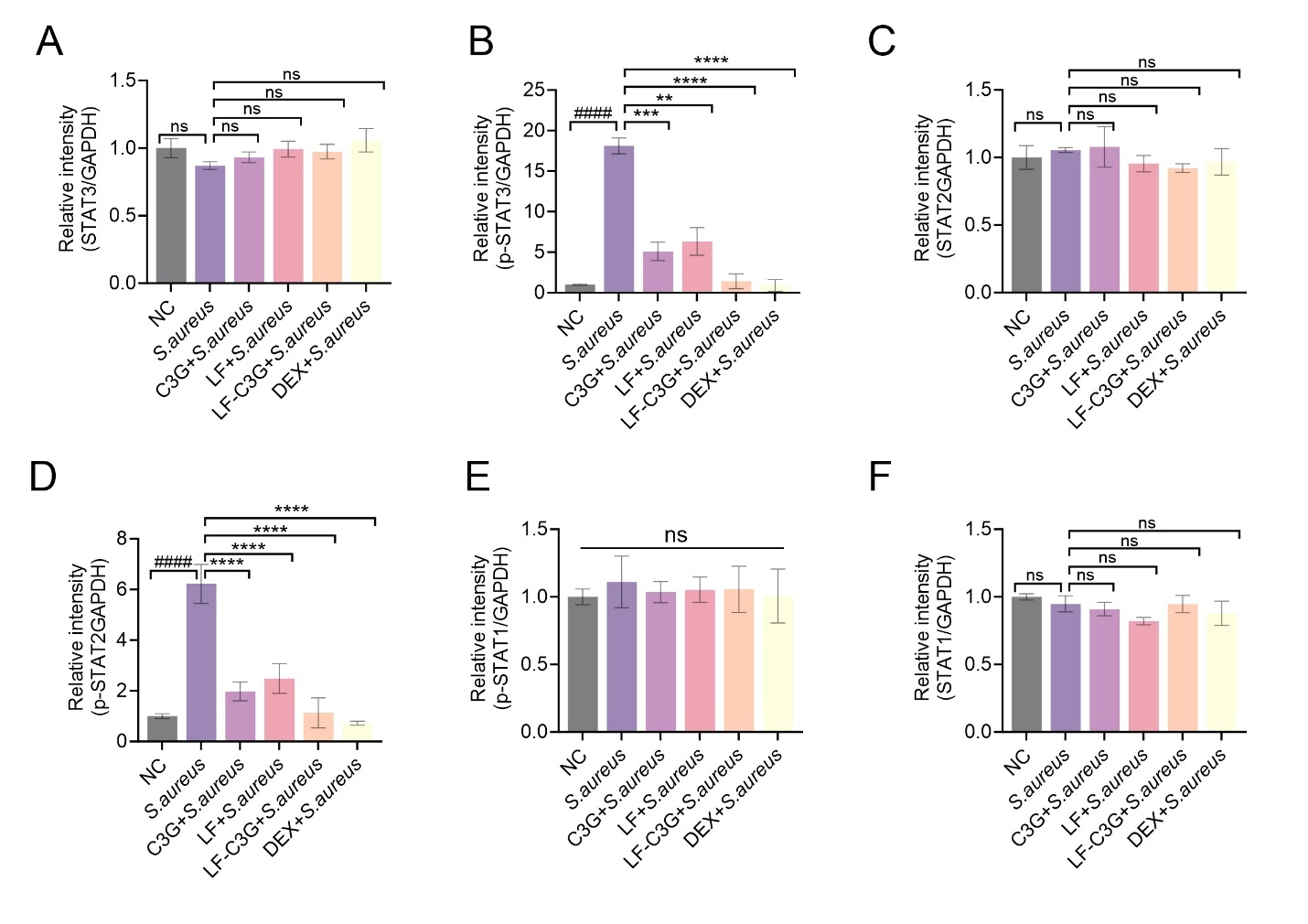


**Figure S5**. (A-F) Quantitative analysis of Phosphorylated STATs (pSTAT1, pSTAT2, and pSTAT3) and STAT1, STAT2, and STAT3 in ***S. aureus*-induced mice** after different treatments. Mean ± SD; **#***p<0.05* compared to the untreated control group; ******p<0.05*, *******p<0.01*, and ********p<0.001* relative to the ***S. aureus***-treated groups.


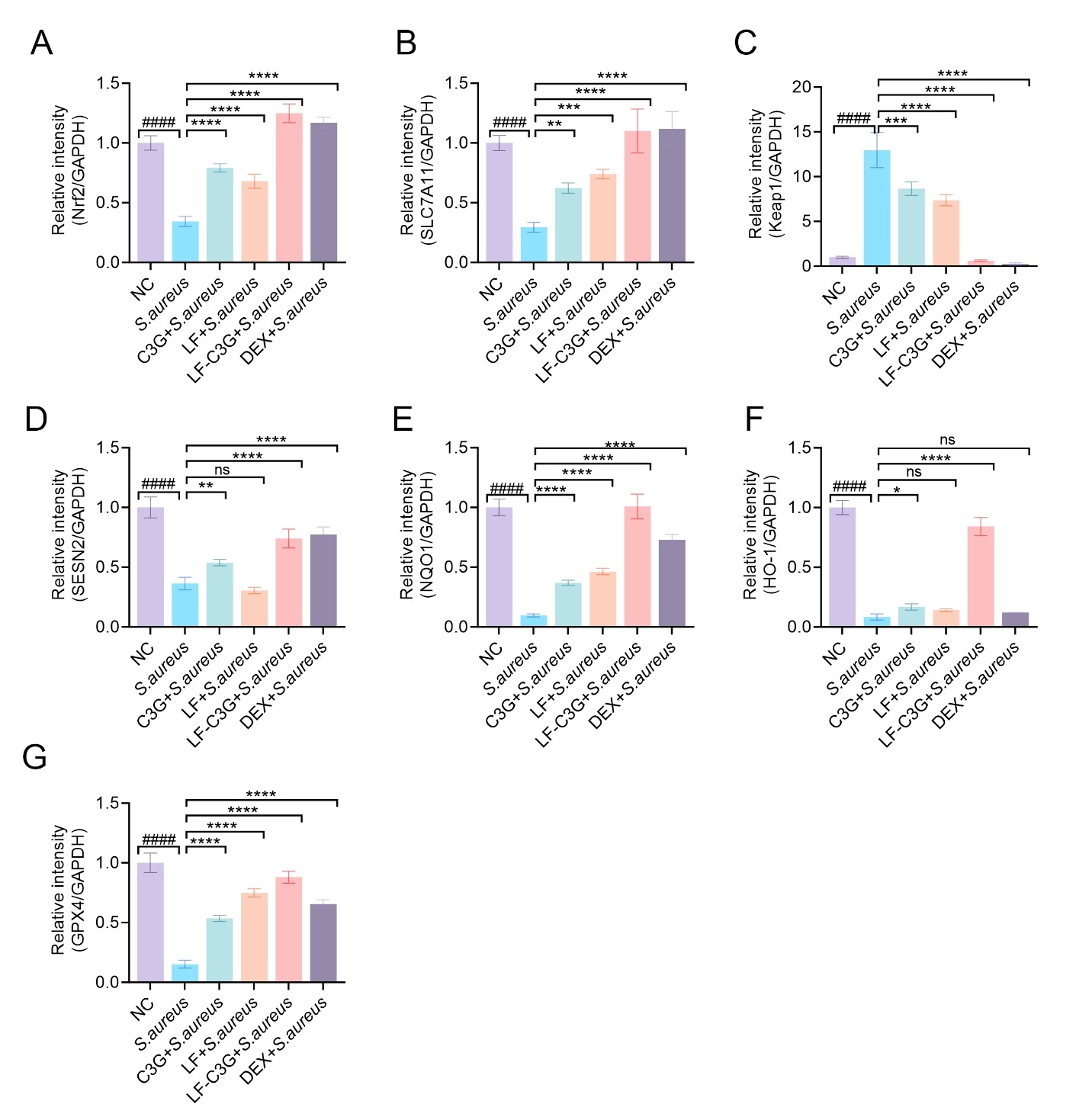


**Figure S6**. Quantitative analysis of Nrf2, Sesn2, Keap1, SLC7A11, NQO1, HO-1, and GPX4 under different treatments. Mean ± SD; **#***p<0.05* compared to the untreated control group; ******p<0.05*, *******p<0.01*, and ********p<0.001* relative to the ***S. aureus***-treated groups.


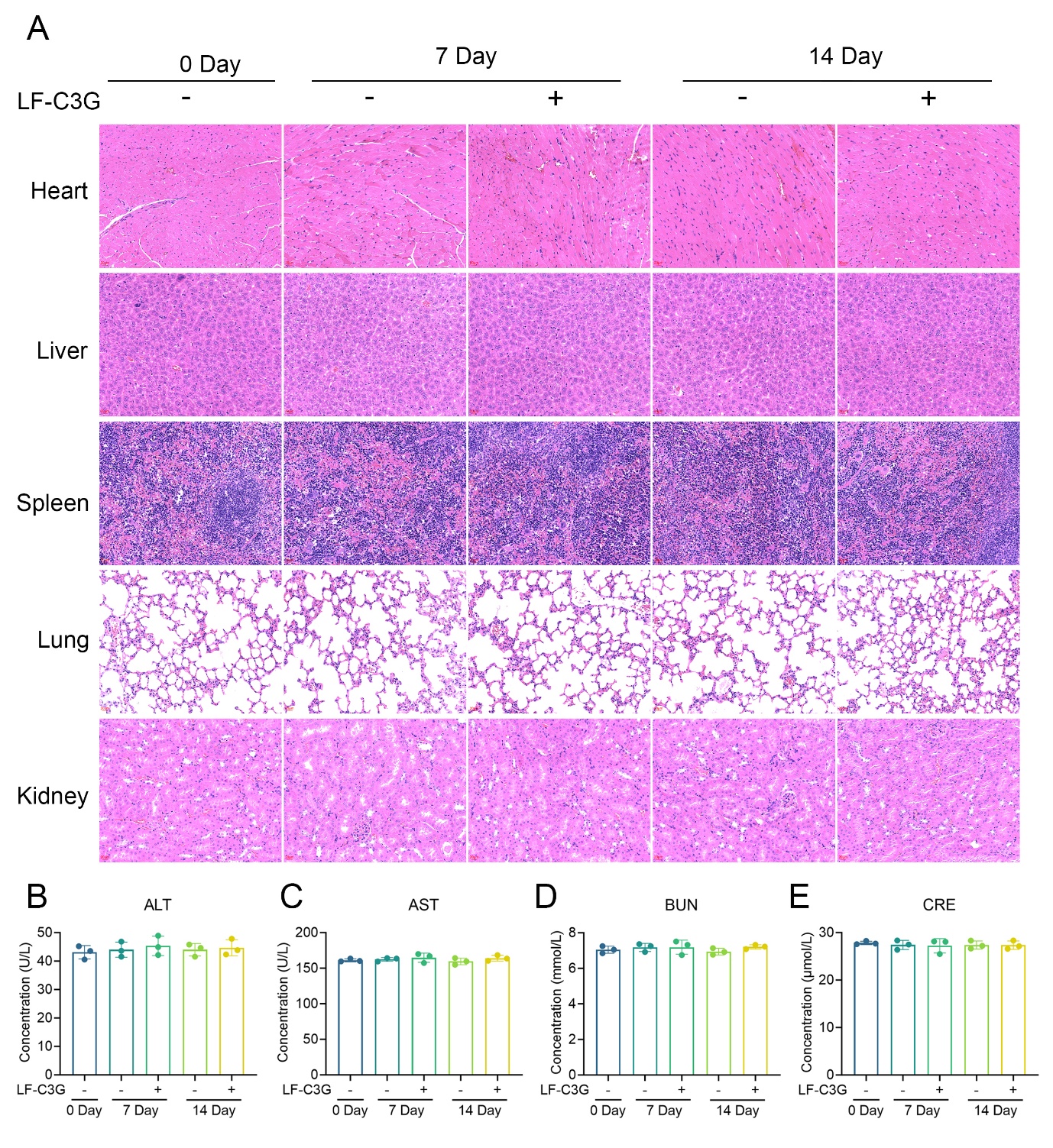


**Figure S7. Evaluation of long-term biosafety of LF-C3GNPs *in vivo*.** (A) Representative H&E-stained sections of major organs (heart, liver, spleen, lung, kidney) from mice treated with LF-C3GNPs for 0, 7, and 14 days. Scale bar, 20 μm. (B–E) Serum biochemical analyses of liver and kidney function, including ALT (B), AST (C), BUN (D), and CRE (E), after LF-C3GNPs treatment for 7 and 14 days. Data are presented as mean ± SD (n = 3).
