# Supplementary material for: Lactoferrin-cyanidin-3-glucoside nanoparticles alleviate inflammation and oxidative stress via Sesn2/Nrf2 activation in mastitis
Source: Mater Today Bio. 2025 Oct 31;35:102491. doi: 10.1016/j.mtbio.2025.102491 (PMC12637076; doi:10.1016/j.mtbio.2025.102491)
Supplement: Multimedia component 2 [file mmc2.docx]

**Tab. S1: The primer sequences for the relative real-time PCR assay.**

| Primer | Nucleotide Sequence (5’-3’) |
| --- | --- |
| GAPDH-F | CCTTCCGTGTCCCTACTGCCAA |
| GAPDH-R | GACGCCTGCTTCACCACCTTCT |
| IL-1β-F | TGGCCCTAAACAGATGAAGT |
| IL-1β-R | GGGAACCAGCATCTTCCTTA |
| IL-6-F | CTGCCTTCCCCGCCCCAGTA |
| IL-6-R | ATGTTACTCCTGTTACATGT |
| TNF-α-F | AGTCAGATCATCTTCTCGAA |
| TNF-α-R | TTCTGATGGCACCACCAGCT |
| MPO-F | GCGCTTCCCCAACGATCAGC |
| MPO-R | TTCTTGATTCGAGGGTCATT |
| SOCS3-F | CTGGTGGTGAACGCCGTGCG |
| SOCS3-R | AAAGCTGCCCCCCTCGCACT |
| SESN2-F | GCTGGGCCTTCACCGGGCCC |
| SESN2-R | GCGAAGGTGCCTGGGAGGCA |
| SLC7A11-F | CTGTGGGCATCACTGTGGTG |
| SLC7A11-R | AAAAAGCCAAGGGCAACCCC |
| Keap1-F | TGGCATCGCCAACTTCGCGG |
| Keap1-R | AGTCGATGCACGCGTGGAAC |
| GPX4-F | GCATCGTCACCAACGTGGCC |
| GPX4-R | TGCCCTTGGGCTGGATCTTC |
| Nrf2-F | GAAGTAGGTAACTGTAGTCC |
| Nrf2-R | AGCTTTGCAAAGTGATAGAT |
| HO-1-F | GGAGATAGAGCGCAACAAGC |
| HO-1-R | GAGCTGCTGGTGGCCCACGC |
| NQO1-F | CGAAGAACTTTCAGTATCCT |
| NQO1-R | GAATGGCTGGCACCCCAAAC |
| STAT1-F | CACGCTGCCAATGATGTTTC |
| STAT1-R | ATCTGTATTGGGTCTTCTTG |
| STAT2-F | CCTGTAATGGAGCCCACACT |
| STAT2-R | GTAAGCCTCATCCACGGTGT |
| STAT3-F | GTGATGCTTCCCTGATTGTG |
| STAT3-R | GCAAGGAGTGGGTCTCTAGG |
